# WERF Endometriosis Phenome and Biobanking Harmonisation Project for Experimental Models in Endometriosis Research (EPHect-EM-Organoids): endometrial organoids as an emerging technology for endometriosis research

**DOI:** 10.1093/molehr/gaaf024

**Published:** 2025-07-09

**Authors:** Elizabeth E Marr, Juan S Gnecco, Stacey A Missmer, Shannon M Hawkins, Kevin G Osteen, Lone Hummelshoj, Erin Greaves, Kaylon L Bruner-Tran, for the EPHect Experimental Models Working Group, Nick A Andrews, Michael S Anglesio, Caroline B Appleyard, Joe Arosh, Christian M Becker, Kaylon L Bruner-Tran, Katherine A Burns, Ronald L Chandler, Julie A Christianson, Fiona L Cousins, Kelsi N Dodds, Victor Fattori, Asgi Fazleabas, Caroline Gargett, Juan S Gnecco, Raul Gomez, Martin Götte, Erin Greaves, Linda G Griffith, Patrick G Groothuis, Ruth Grümmer, Sun-Wei Guo, Shannon M Hawkins, M Louise Hull, Lone Hummelshoj, Mark Hutchinson, Mohamed Gamal Ibrahim, Elizabeth E Marr, Stacy L McAllister, Stacey A Missmer, Jeffrey Mogill, Jens Nagel, Warren B Nothnick, Paulina Nunez-Badinez, Kevin G Osteen, Daniëlle Peterse, Michael S Rogers, Andrea Romano, Philippa T K Saunders, Miguel Ángel Tejada, Kathy L Sharpe-Timms, Waldiceu A Verri, Paola Viganó, Katy Vincent

**Affiliations:** Department of Biomedical Engineering, Tufts University, Medford, MA, USA; Department of Biomedical Engineering, Tufts University, Medford, MA, USA; Mother Infant Research Institute, Tufts Medical Center, Boston, MA, USA; Department of Epidemiology, Harvard T.H. Chan School of Public Health, Boston, MA, USA; Department of Obstetrics and Gynecology, University of Michigan, Ann Arbour, MI, USA; World Endometriosis Research Foundation, London, UK; Department of Obstetrics and Gynecology, Indiana University School of Medicine, Indianapolis, IN, USA; Department of Obstetrics and Gynecology, Vanderbilt University Medical Center, Nashville, TN, USA; World Endometriosis Research Foundation, London, UK; World Endometriosis Research Foundation, London, UK; Division of Biomedical Sciences, Warwick Medical School, University of Warwick, Coventry, UK; World Endometriosis Research Foundation, London, UK; Department of Obstetrics and Gynecology, Vanderbilt University Medical Center, Nashville, TN, USA

**Keywords:** endometriosis, experimental models, organoids, emerging models, research, collaboration

## Abstract

The aetiology of endometriosis remains poorly understood. *In vitro* model systems provide the opportunity to identify the mechanisms driving disease pathogenesis using human cells. Three-dimensional models, particularly organoid systems, have revolutionized how we study epithelial biology and are powerful tools for modelling endometriosis. As an emerging model system, it is important to define protocols and identify the remaining challenges surrounding endometrial organoid culture to increase reproducibility and scientific rigour in endometriosis research. The World Endometriosis Research Foundation (WERF) established an international working group comprised of experts using *in vitro* approaches for the study of endometriosis. This working group harmonized protocols and documentation of existing and emerging organoid systems to maximize comparison and replication across the field and guide specific research hypotheses testing. This evaluation of organoid protocols, limitations, challenges, and alternative approaches assessed both published and grey literature papers across several disciplines pertinent to endometriosis research. Recommendations for protocol and documentation harmonization are presented, and we created the first-ever decision tree diagram to guide and facilitate the selection of existing models best suited for specific areas of endometriosis research. Rigorous and systematic assessment of emerging organoid systems, recognizing the inferential strengths and limitations of these approaches, is vital for endometriosis research. This comprehensive review of the benefits, limitations, and utilization of organoid models, as well as the consequent integration of protocols and documentation, will contribute to the scientific knowledge base by maximizing the reproducibility, comparability, and interpretation of research studies in endometriosis. Additionally, these newly developed protocols and documentation should serve as a resource for, and facilitate collaboration between, endometriosis investigators using organoids in their research methods.

## Introduction

Endometriosis affects an estimated 190 million women (and those assigned female at birth) worldwide, with a significant personal and societal burden due to its painful and fertility-related symptoms (Nnoaham *et al.*, 2011; Zondervan *et al.*, 2020). There is no known cure, and treatments are associated with low long-term success rates and significant side effects (Johnson *et al.*, 2013). Endometriosis is defined as the growth of endometrial-like tissue outside the uterus; however, this definition does not encompass the complex symptomatic, pathobiologic, and multisystemic nature of the disease (Zondervan *et al.*, 2020). The overwhelming prevalence of endometriosis and the unknown aeteology highlight the need for a better understanding of the basic biology of endometriosis formation and persistence to enable subsequent identification of targets for effective therapies for endometriosis.

Endometriosis lesions are complex multicellular tissue deposits composed of endometrial-like stromal cells with or without epithelial cells or glands embedded within a microenvironment of extracellular matrix, fibrosis, scarring, and sometimes haemorrhage (Clement, 2007; Vigano *et al.*, 2018). Additionally, lesions are infiltrated by blood vessels, nerve fibres, and immune cells, contributing to a highly inflammatory setting (Greaves *et al.*, 2017; Forster *et al.*, 2019; Hogg *et al.*, 2020, 2021; Panir *et al.*, 2022). The histological characteristics of lesions vary widely, with differences in the presence and extent of endometrial-like cells (Zondervan *et al.*, 2020), fibrosis (Li *et al.*, 2022), and immune or nerve infiltration (Tran *et al.*, 2009). These variations suggest that the pathological processes within each lesion are dynamic and may or may not fluctuate in synchronicity with the menstrual cycle (Nnoaham *et al.*, 2011). This could help explain the diverse clinical presentation, variable responses to treatment, and the evolving nature of lesions, which are often distinguished by deposits of different colours at various stages of their development (Zondervan *et al.*, 2020). Thus, experimental models are an essential tool to mechanistically dissect the pathophysiology of this complex disorder and identify novel treatment targets. While animal models of endometriosis are important to study the establishment, progression, and regression of endometriosis via the surgical induction of disease (Bruner *et al.*, 1995; Grümmer, 2012; Burns *et al.*, 2025; Dodds *et al.*, 2025; Hull *et al.*, 2025), most of these experimental animals do not naturally develop this disease and limit translational potential to the human condition. Therefore, the development of humanized *in vitro* model systems provides a mechanistic approach to dissect the underlying complexity of endometriotic disease.

The lack of menstrual shedding and the immune differences in animal models of endometriosis limit the translational potential to human applications. However, traditional *in vitro* models utilizing two-dimensional (2D) human cell culture methods suffer from an overwhelmingly simple and non-physiological context. Recent advances in three-dimensional (3D) tissue platforms, particularly the development of epithelial organoid technologies and associated organoid systems (i.e. multi-cellular organoid-derived models), offer an opportunity to revolutionize the investigation of both the eutopic endometrial epithelium and ectopic endometriotic lesions. The emergence of endometrial epithelial organoid (EEO) systems represents an exciting and innovative technology for research in reproductive biology. To maximize the potential of this model, it is imperative to identify the existing challenges, harmonize protocols, and recognize both the limitations and potential that organoids offer to help advance endometriosis research within the field. Herein, we identify key considerations when working with endometrial organoids with the goal of increasing experimental reproducibility and scientific rigour. Additionally, we discuss key experimental factors to consider when conducting endometriosis-associated research with organoids. By highlighting the benefits and limitations of discovery efforts using *in vitro* platforms, we provide a guide for those already in, or considering entering, the endometrial organoid space, as well as important considerations for investigators trying to find the right model for their research questions.

Recommendations and SOPs were also developed for heterologous rodent models, homologous rodent models, and endometriosis pain models, and the results are presented in the associated EPHect companion papers (Hull *et al.*, 2025; Burns *et al.*, 2025; and Dodds *et al.*, 2025, respectively).

## Methods

The World Endometriosis Research Foundation (WERF) Endometriosis Phenome and Biobanking Harmonisation Project (EPHect) previously established standardized recommended and minimum requirements for the collection of clinical and surgical data from individuals with endometriosis (Becker *et al.*, 2014; Vitonis *et al.*, 2014). Additionally, WERF has developed a standardized physical examination assessment for endometriosis (Lin *et al.*, 2024) and internationally agreed-upon standard operating procedures (SOPs) for the collection, processing, and storage of tissue and fluid biospecimens (Fassbender *et al.*, 2014; Rahmioglu *et al.*, 2014). As part of this effort, WERF recognized a critical gap in the development of standardized protocols for emerging organoid models for endometriosis. The objective was to evaluate these models for their ability to replicate key disease hallmarks to facilitate the reliable visualization and analysis of lesion modelling outcomes. Following a number of workshops, internationally renowned researchers, with expertise in *in vitro* modelling of endometriosis, were invited to join the EPHect Experimental Models Working Group (Supplementary Fig. S1). Working group members were identified based on pioneering work of organoid model(s) for endometrium and endometriosis. For identification of publications, the search criteria were as follows: ‘endometriosis’ + ‘organoid’ + ‘endometrium’. We subsequently used an artificial intelligence-based research platform, Research Rabbit, into which we input landmark endometrial organoid papers to assist in the unbiased identification of related endometrial organoid models and publications. The aims of this article were to reappraise the current and emerging *in vitro* models for the study of endometriosis and to develop SOPs that meet a minimum set of standards based on the objective(s) of the study (decision tree). All models were examined with the understanding that an individual model is unlikely to address all parameters of endometriosis and instead the focus was on identifying research standards critical for the use of organoid model, and their limitations, in the study of endometriosis.

## Results

### Organoid technologies for endometrial epithelial biology

The study of endometrial epithelium through traditional 2D cell culture methods has historically been a major technical challenge, resulting in study design limitations that impact reproducibility within and between research groups. Primary endometrial epithelial cells cultured on hard plastic (i.e. polystyrene) dishes rapidly undergo de-differentiation (Zhou *et al.*, 2022) and, in the presence of serum, further lose physiological attributes (Ghosh and Sengupta, 1995) and epithelial barrier integrity, and have increased cellular senescence by the third cell passage (Arnold *et al.*, 2001; Wang *et al.*, 2012; Li *et al.*, 2018). Specifically, endometrial epithelial cells cultured in 2D monolayers undergo significant cellular flattening, lose their pseudostratified architecture, and create sparse colonies of cells with reduced functionality (Hennes *et al.*, 2019). The development of 3D organoid technologies overcomes many of these challenges by providing a robust and physiologic approach to study the endometrial epithelia *in vitro*.

An ‘organoid’ is a self-organizing organotypic 3D tissue construct that mimics the architecture and the function of its corresponding *in vivo* tissue source. In uterine biology, the earliest use of the term ‘organoid’ dates back to the late 1980s, when isolated endometrial gland fragments were embedded in an extracellular matrix protein, such as Matrigel or collagen (Rinehart *et al.*, 1988; Bentin-Ley *et al.*, 1994) to establish glandular structures *in vitro*. However, it was the work pioneered by Hans Clevers’ group in the early 2000s (Sato *et al.*, 2009, 2011) that standardized the protocols and methodology to generate organoids from numerous tissues. Borrowing from the paradigm in tissue engineering depicting the integration of cells, signalling cues, and biomaterials, they defined a method to reproducibly generate epithelial organoids by providing the necessary cues to stimulate the stem and progenitor cell populations to promote the generation of these tissue-like constructs. Their organoid methodology has since been used to develop *in vitro* models of both health and disease in numerous tissues (Clevers, 2016). Several studies have demonstrated the utility in leveraging organoids to examine human physiology in health and associated diseases, including liver organoids to study fibrosis (Leite *et al.*, 2016), hepatitis (Fu *et al.*, 2019), and non-alcoholic fatty liver disease (Kruitwagen *et al.*, 2017); lung organoids to investigate pulmonary infection and inflammation (Salahudeen *et al.*, 2020); and intestinal organoids to elucidate mechanisms of rotovirus infection (Zou *et al.*, 2019). Thus, the generation of organoids from the endometrium enables the study of elusive mechanisms driving epithelial biology in reproductive health.

**Figure 1.**
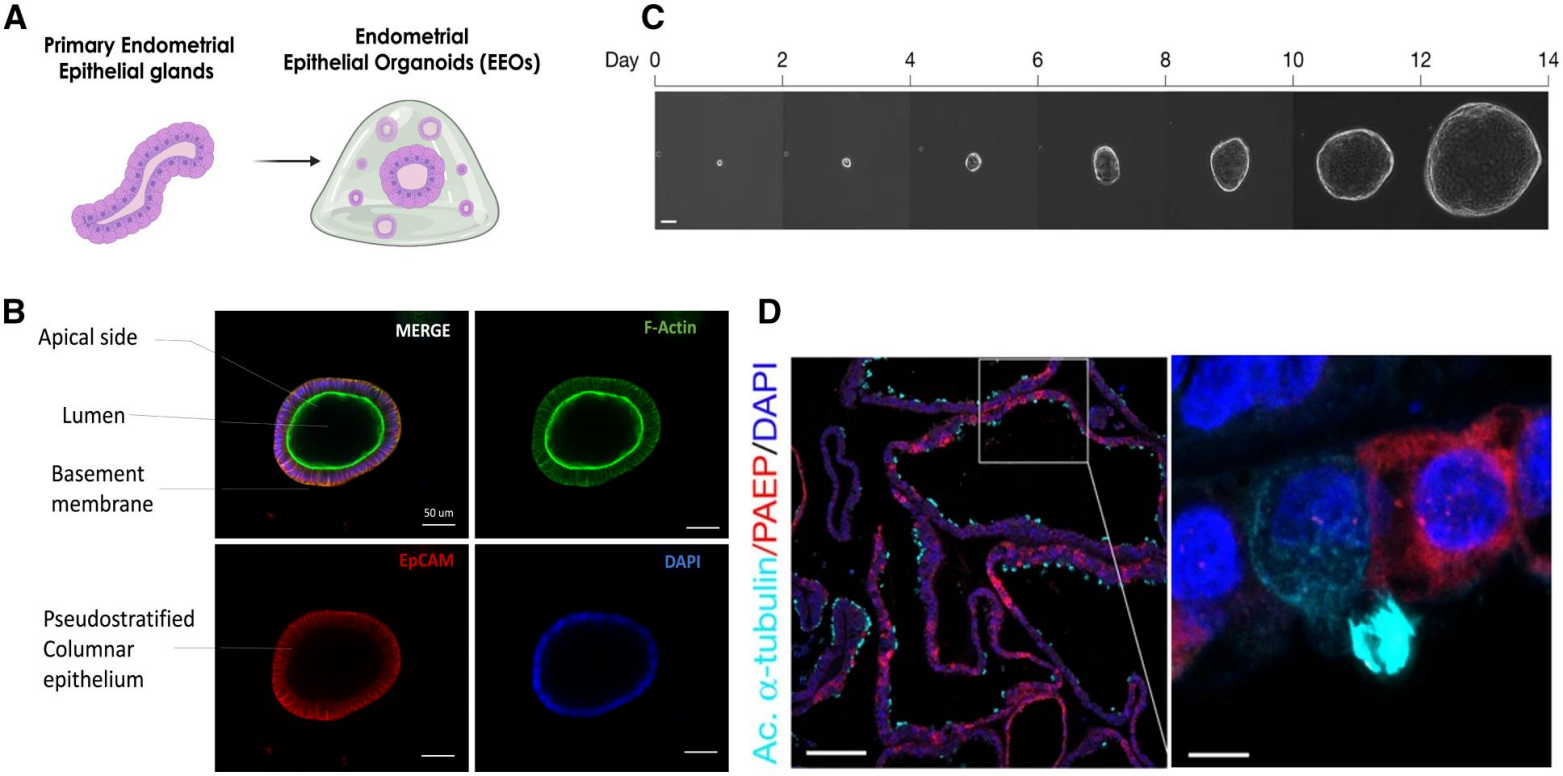
**Characterization of endometrial epithelial organoids (EEOs).** EEOs are *in vitro* gland-like structures generated from adult-derived primary endometrial epithelial glands. (**A**) Schematic of the generation of organoids from endometrial glands. (**B**) Immunofluorescent staining confirms the epithelial phenotype of EEOs, with epithelial marker EpCAM (red) staining organoid epithelial cells, while F-actin (green) staining highlights the polarization and architecture of pseudostratified columnar epithelial cells. Scale bar = 50 μm. (**C**) Time-lapse imaging of EEOs derived from a single cell across 14 days of culture. EEOs are clonal and can be maintained in 3D hydrogel cultures for multiple weeks to generate multicellular structures. Scale bar = 500 μm. (**D**) EEOs stain positively for secretory markers such as progestogen-associated endometrial protein (PAEP), with secretory cells that will secrete into the luminal centre of the organoid. EEOs also stain positively on the luminal cell surface of polarized epithelial cells for cilia via acetylated α-tubulin (Ac. α-tubulin). Scale bars = 100 μm (left) and 5 μm (right). Panels B and C were adapted from Turco *et al.* (2017), Fig. 5a (cropped) and Fig. 5c, respectively (Turco *et al.*, 2017) used by permission under a Creative Commons Attribution License CC BY (2012).

In 2017, two landmark papers established the first protocols to generate epithelial organoids of the human endometrium (Boretto *et al.*, 2017; Turco *et al.*, 2017). Establishing organoids from adult tissues relies on stimulating the adult-stem and/or progenitor cell populations to promote the mitogenic and differentiation processes leading to epithelial expansion and maturation, respectively (Sato *et al.*, 2009). Using primary endometrial tissues from patient biopsies, EEOs are generated by embedding epithelial endometrial gland fragments in a hydrogel scaffold (e.g. Matrigel) and stimulating the cells with a defined serum-free medium comprised of a cocktail of growth factors and inhibitors designed to enrich the stem/progenitor cell populations (Supplementary File S1). This approach generates *in vitro* gland-like analogues that mimic epithelial gland morphology and function (Fig. 1A). EEOs are clonally derived and comprised of a hollow sphere made up of a monolayer of heterogeneous pseudostratified epithelium (Fig. 1B) with a biochemically selective barrier (Simintiras *et al.*, 2021). Specifically, EEOs demonstrate proper cell polarity, meaning that the cytoskeletal microvilli and cilia, normally exposed to the luminal surface, are found in the inside of the hollow sphere they form with external deposition of basement membrane (BM) proteins on the outside of the sphere (Fig. 1C). In addition to their architecture, EEOs faithfully mimic *in vivo* epithelial gland function, recapitulating epithelial subpopulation heterogeneity (Fig. 1D), including the secretory and ciliated epithelia, maintaining their genetic stability across passages and cryopreservation (Boretto *et al.*, 2017; Turco *et al.*, 2017), and expressing oestrogen receptor (ESR) and progesterone receptor (PGR), thereby retaining sex hormone responsive phenotypes (e.g. progestogen-associated endometrial protein (PAEP) secretion in response to progesterone treatment) over multiple passages (Boretto *et al.*, 2017).

The generation, expansion, propagation, and manipulation of EEOs as a model for the endometrial epithelium have the potential to revolutionize the reproductive health space. This technology overcomes major historical limitations that have hindered advances in the reproductive field by: (i) facilitating the ability to reproduce studies using primary epithelial cells through cryopreservation and biobanking, (ii) enabling the study of physiologically relevant populations and tissue-like structures that capture cellular polarity, architecture, and hormonal function, and (iii) providing the opportunity to investigate patient-specific phenotypes in controlled *in vitro* systems. To date, EEOs have already been used to study the functional composition and mechanisms regulating epithelial differentiation and heterogeneity (Fitzgerald *et al.*, 2019; Haider *et al.*, 2019; Luddi *et al.*, 2020). For example, by stimulating EEOs with oestradiol and pharmacologic inhibitors, Haider *et al.* (2019) demonstrated a molecular mechanism whereby the coordinating crosstalk between oestrogen and NOTCH signalling regulates ciliogenesis in the endometrium (Haider *et al.*, 2019). Furthermore, EEOs have also been instrumental in studying the crosstalk between the blastocyst and the endometrium to identify a potential role of mechanosensitive ion channels in blastocyst–endometrial communication through the functional measurement of calcium signalling (Hennes *et al.*, 2019). Finally, the generation of rodent-derived endometrial organoids from genetically engineered mice has also advanced the investigation of elusive pathways that have previously been challenging to study in other *in vitro* systems. These include studying the role of Wnt signalling and Lgr5+ cells in the murine endometrium for endometrial regeneration (Seishima *et al.*, 2019); advancing models to study the pathogenic colonization mechanisms of *Chlamydia trachomatis* infections in the endometrial epithelia (Bishop *et al.*, 2020; Dolat and Valdivia, 2021); and investigating the role of KRAS mutations in endometrial cancer development (Maru *et al.*, 2019; 2021). Although research with human endometrial organoids remains an emerging technology and has, to date, mainly focused on the eutopic endometria, it is poised to advance the investigation of the mechanisms associated with the initiation and progression of endometriosis (Boretto *et al.*, 2019).

### Key considerations for working with organoid technologies

#### Organoid nomenclature

A first point of harmonization for endometrial organoid technologies is the associated nomenclature. Rinehart *et al.* first used the term ‘organoid’ to describe the spontaneous outgrowth of endometrial epithelial glands cultured in Matrigel (Rinehart *et al.*, 1988), which served to help build early iterations of *in vitro* co-culture models (Arnold *et al.*, 2001; Bläuer *et al.*, 2005). However, the current term ‘organoids’ is most associated with the work of Turco *et al.* (2017) and Boretto *et al.* (2017), whereby they defined an organoid as a 3D self-assembling tissue construct derived from adult stem/progenitor cells that mimic the architecture and function of the tissue based on the Clevers’ and Sato’s original protocols (Clevers, 2016). However, the term ‘organoids’ has also been used to describe other organotypic models, including those derived from pluripotent or cellular aggregates of somatic cells using scaffold-free micro-moulded agarose wells (Wiwatpanit *et al.*, 2020; Song and Fazleabas, 2021). Such cell aggregate models (Białkowska *et al.*, 2020; Stejskalová *et al.*, 2021) rely on multi-cellular aggregation rather than the generation of hollow epithelial structures derived from stem/progenitor cells (Clevers, 2016; Boretto *et al.*, 2017; Deane *et al.*, 2017; Kim *et al.*, 2020; Turco *et al.*, 2017). This lack of consensus may lead to confusion when comparing studies that use organoid models. Furthermore, across the literature, we find that the acronym used for EEOs also varies by research group and includes terms such as ‘endometrial glandular ORG (ORG)’ (Luddi *et al.*, 2021), ‘endometrial organoid (EMO)’ (Boretto *et al.*, 2017), ‘ectopic organoid (ECT-O)’ (Boretto *et al.*, 2019), ‘endometrial epithelial organoid (EEO)’ (Fitzgerald *et al.*, 2019; Hernandez-Gordillo *et al.*, 2020; Cheung *et al.*, 2021; Gnecco *et al.*, 2023), and ‘endometrial organoids (EORG)’ (Haider *et al.*, 2019), which may mislead interpretation across the different studies. We propose that those models derived from the endometrial epithelial glands, using the paradigm set by Clevers and honed for tissue specificity by Turco *et al.* and Boretto *et al.*, are to be considered true endometrial ‘organoids’, while aggregate models that yield 3D structures are more appropriately referred to as ‘spheroids’. In this review, when referring to endometrial epithelial organoids, we will use the term ‘EEO’, as it is the most used acronym in the literature and accurately describes its endometrial epithelial derivation. The incorporation of multiple cell types within organoid models will be discussed in more detail later, using terms such as ‘multi-cellular organoid systems’ or ‘assembloids’ (Rawlings *et al.*, 2021) to capture the complex nature of these multi-cellular cultures.

#### Cell and tissue sourcing

A second point of harmonization is the need to define cell and tissue sourcing for the generation of EEOs. Historically, cell lines have been routinely used to study endometriosis. These cell lines include the immortalized 12Z epithelial and 22B stromal lines derived from the endometrial peritoneum of a 37-year-old female undergoing laparoscopy and immortalized using SV40 plasmid transfection (Zeitvogel *et al.*, 2001). These cells capture some of the hallmarks of endometriosis, such as over-secretion of the pro-inflammatory cytokine interleukin 1 beta (IL-1 β) (Mori *et al.*, 1991), altered hormone sensitivity (Patel *et al.*, 2017), increased prostaglandin and matrix metalloprotease (MMP) secretion (Banu *et al.*, 2008), and a more invasive phenotype (Zeitvogel *et al.*, 2001; Stejskalová *et al.*, 2021) compared to primary endometrial epithelial and stromal cells isolated from the eutopic endometrium. These cell lines are often employed as tools for building models of endometriosis using scaffolds for multi-cellular cultures due to their commercial accessibility (Banu *et al.*, 2009; Brueggmann *et al.*, 2014; Wendel *et al.*, 2020; Stejskalová *et al.*, 2021). Despite their convenience, these immortalized cell lines do not fully capture the heterogeneous phenotype of cells from patient-derived tissues. The advent of endometrial organoids provides a more physiological and clinically relevant model for studying endometriosis by growing cells directly from patient populations and their ectopic tissues.

**Figure 2.**
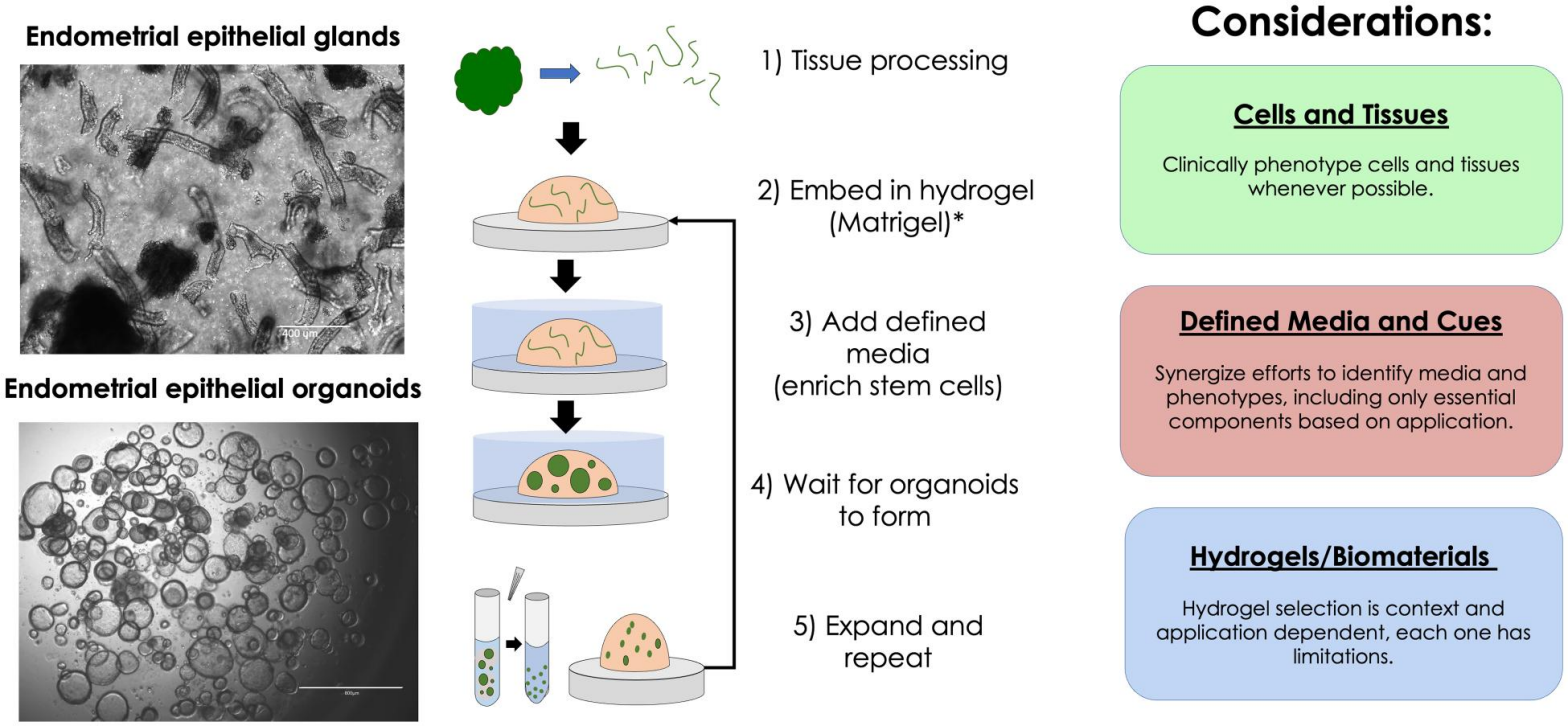
**Principles and key steps in the generation of endometrial epithelial organoids (EEOs).** Schematic of the generation of endometrial organoids. EEOs are sourced from primary endometrial epithelial glands, embedded in Matrigel* (or another 3D matrix) and then supplemented with defined media to support organoid formation and growth. Organoids can be enzymatically and mechanically digested to single cells then passaged, and the process can be repeated to build libraries of EEOs from various donors. The scale bars in images represent 400 μm (top) and 800 μm (bottom).

EEOs are derived from adult primary endometrium, thereby providing an opportunity for clinical translation using patient-derived endometrial and endometriotic-diseased tissues. Through well-established protocols for endometrial isolations (Osteen *et al.*, 1989; Romano *et al.*, 2020, 2022), glandular epithelia populations can be obtained to generate organoid cultures that are suitable for clonal expansion and cryopreservation (Fig. 2). To date, the generation of EEOs has primarily been derived from the eutopic endometria of tissue donors, including patients with and without endometrial pathologies such as endometriosis (Boretto *et al.*, 2019), infertility (Bui *et al.*, 2020), and adenomyosis (Juárez-Barber *et al.*, 2022). Nonetheless, it is important to recognize that the use of primary cells presents several challenges that may impact scientific rigour and reproducibility. Variability in clinical phenotype, cycle phase, inherent patient heterogeneity, and cell isolation purity are parameters that can add variability to patient-sourced endometrial specimens (Romano *et al.*, 2020). Therefore, robust clinical phenotyping and complete metadata (e.g. cycle phase, demographics, age, drug exposure) are essential to accurately interpret study results and should be clearly reported. Finally, it should be recognized that access to primary tissues may not be equitable for all research groups and usually requires strong partnerships with clinical investigators. However, one advantage of EEOs is their clonal generation, meaning that from a small initial sample, even from a single cell (Fig. 1C), EEO lines can be generated to enable robust, stable, and continuous expansion for numerous passages and timeframes. Moreover, EEOs can also be generated from non-invasive means, including menstrual effluent (menstrual organoids), which retain the same stability and phenotype as those isolated from fresh tissue biopsies, including hormone sensitivity (Cindrova-Davies *et al.*, 2021). Finally, EEOs can also be generated from cryopreserved endometrial tissues, which are functionally and genetically comparable to matched fresh tissue-derived organoids (Bui *et al.*, 2020; Heidari-Khoei *et al.*, 2022). These non-invasive alternatives for tissue sourcing of endometrial tissues, compared to fresh endometrial biopsies, should enable clinically restricted research groups greater access to patient-derived cells. These approaches provide solutions for challenges associated with downstream processing and tissue transport. We propose the establishment of a centralized endometrial organoid biobank of clinically phenotyped endometrial and endometriotic organoids may be a tractable and necessary effort to facilitate access to organoids for the larger endometriosis research community.

#### Specialized media

A third point of unification is the composition of the media needed for generating organoids. The generation of organoids across multiple mucosal tissues (Sato *et al.*, 2009, 2011; Boretto *et al.*, 2017; Turco *et al.*, 2017) requires a specialized media comprised of growth factors and inhibitors designed to stimulate the stem/progenitor cell populations. In the intestine organoid model, and mirrored in other tissue organoids, the importance of Wnt/β-catenin pathway stimulation has proved imperative for maintaining long-term 3D culture via the enrichment of progenitor cell populations (Clevers, 2016). Indeed, early efforts of endometrial organoid formation relied heavily on both Wnt and R-spondin proteins in media formulations (Boretto *et al.*, 2017; Turco *et al.*, 2017); however, their requirement depended on the species of source tissue the EEOs were generated from (e.g. murine or human). The role of specific recombinant proteins, supplements, pathway inhibitors, and hormones in organoid media is discussed below and summarized in Table 1 and Supplementary Table S1 to recapitulate the important growth media parameters to consider when using 3D endometrial organoid cultures.

**Table 1.** Media components commonly utilized for the sustained culture and hormone-initiated differentiation of endometrial organoid systems (EEOs).

			EEOs							EEOs + stroma	
Category	Reagent	Primary use/notes	Shared	Turco *et al.* (2017)	Boretto *et al.* (2017)	Boretto *et al.* (2019)	Hernandez-Gordillo *et al.* (2020)	Francés-Herrero *et al.* (2021)	Garcia-Alonso *et al.* (2021)	Rawlings *et al.* (2021)	Gnecco *et al.* (2023)
*Base media*	Advanced DMEM/F12			√				√			
	DMEM/F12	With phenol red: B, C, E; phenol-free: D			√	√	√		√	√	√
*Conditioned media*	R-spondin-1 media	Used in place of recombinant R-spondin			√	√					
	Wnt3A media	Used in place of recombinant Wnt3A			√						
*Recombinant proteins*	Recombinant human EGF	Epidermal growth factor	√	√	√	√	√	√	√	√	√
	Recombinant human bFGF/FGF-2	Fibroblast growth factor-2, also known as FGFbasic				√					
	Recombinant human FGF-10	Fibroblast growth factor-10	√	√	√	√	√	√	√	√	√
	Recombinant human HGF	Hepatocyte growth factor		√				√	√	√	
	Recombinant human Noggin	Bone morphogenetic protein 4 antagonist	√	√	√	√	√	√	√	√	√
	Recombinant human Rspondin-1	Wnt/β-catenin signalling pathway stimulator		√			√	√	√	√	√
*Supplements*	B27	with or without vitamin A, Gnecco made in house without P4	√	√	√	√	√	√	√	√	√
	EndoECM	0.01 mg/ml used as a soluble additive in stripped down media						√			
	Insulin–transferrin–selenium	supports metabolic pathways and mitigates ROS	√*	*	√	√	√		√	√	√
	L-glutamine/glutamax	Amino acid	√	√	√	√	√	√	√	√	√
	N2	Gnecco made in house without P4	√	√	√	√	√	√	√	√	√
	N-acetyl-L-cysteine	Antioxidant and mucolytic agent	√	√	√	√	√	√	√	√	√
	Nicotinamide	Supplement amide derivative of vitamin B3 and a PARP inhibitor	√	√	√	√	√	√	√	√	√
*Inhibitors*	A83-01	TGF-β/ALK pathway inhibitor	√	√	√	√	√	√	√	√	√
	SB202190	p38 inhibitor			√	√			√		
	Y-27632	Rock inhibitor only used temporarily after passage			√	√	√				
*Antibiotic/antifungal*	Penicillin/streptomyocin	Antibiotic, antifungal			√	√	√				√
	Primocin	Antibiotic, antifungal		√				√	√		
*Hormones*	17-β oestradiol (E2)	↑ proliferation, ESR expression	√	√	√	√	√		√	√	√
	8-Bromoadenosine 3'5'-cyclic monophosphate (cAMP)	w/P4 accelerates ↑ glandular phenotype, ciliation		√						√	
	hCG	w/hPL + cAMP, ↑ glandular phenotype, ↓ proliferation		√							
	Human placental lactogen (hPL)	w/hCG + cAMP, ↑ glandular phenotype, ↓ proliferation		√							
	Medroxyprogesterone acetate (MPA)	↑ Glandular phenotype, ↓ PGR expression, proliferation					√			√	√
	Progesterone (P4)	↑ Glandular phenotype, ↓ PGR expression, proliferation		√							
	Prolactin (PRL)	Secreted by decidualized stromal cells		√							

Comparison of organoid expansion media components used in organoid research shared across publications working with EEOs. *Included in formula of Advanced DMEM/F12 media.

#### Base media

The foundational base of EEO media is a commercially available phenol-free 1:1 mixture of Dulbecco’s Modified Eagle’s Medium (DMEM) and Ham’s F12 media (F12), referred to as DMEM/F12, a base medium routinely used for reproductive cell culture. A modified formula of DMEM/F12, known as Advanced DMEM/F12, has also been used in a study performed by Turco *et al.* (2017), which contains additional supplements that are added exogenously when using DMEM/F12. These additional components include super-physiologic glucose concentrations, ascorbic acid phosphate, AlbuMAX™ II, human transferrin, insulin, glutathione, ammonium metavanadate, manganous chloride, sodium selenite, and ethanolamine (ThermoFisher, Waltham, MA, USA). Presumably, by using Advanced DMEM/F12, Turco *et al.* omitted the inclusion of insulin–transferrin–selenium cocktail, which was not included in their final organoid media composition (Table 1). A notable difference between DMEM/F12 and Advanced DMEM/F12 is the inclusion of the pH indicator phenol red. The inclusion of phenol red in Advanced DMEM/F12 is important due to its structural resemblance to oestradiol and ability to confer oestrogenic properties by binding to oestrogen receptors (Berthois *et al.*, 1986). Regular DMEM/F12 is available either with or without the addition of phenol red; however, Advanced DMEM/F12 does not have a commercial option in which phenol red is omitted. The presence of phenol red has been shown to signal as an oestrogen mimic with a relative activity on par with oestrogens in the 15–45 µM concentration range when cultured with human breast cancer cells (Berthois *et al.*, 1986). Given the sensitivity of reproductive tissues to oestradiol, using a phenol red-free medium with a defined concentration of oestrogen is highly recommended for endometrial *in vitro* studies.

#### Serum-free growth medium supplements

EEOs require media conditions that are serum-free. Due to the lack of foetal bovine serum (FBS) in the generation of EEOs, the basal expansion media contains additional supplements that support organoid growth in lieu of serum. These supplements include amino acids, vitamins, and antioxidants, which provide the necessary building blocks for general cell growth and maintenance. First, N-2 and B-27 are commercial supplements commonly made up of cocktails of 5 and 21 respective components originally derived for use in stem cell cultures (ThermoFisher). N-2 and B-27 were originally developed for neuronal *in vitro* cultures (Brewer *et al.*, 1993; Brewer, 1995); however, the mechanisms regarding their influence on cellular homeostasis are not fully understood. Both supplements are present in all the media reported in this article (Table 1) and are routinely used in other tissue organoid cultures. Relevant to endometriosis research, it is important to recognize that progesterone (2 μM in N-2 and an undisclosed concentration for B-27) is a component of these supplement cocktails, which may interfere with sex hormone responses in endometrial organoids (Hewitt *et al.*, 2022). L-glutamine is an amino acid that is added to cell culture media for maintaining cellular processes, including the production of proteins and glucose. An alternative to L-glutamine, GlutaMAX™, is often used in organoid media (ThermoFisher). *N*-acetylcysteine (NAC), a derivative of the amino acid L-cysteine added to all EEO media discussed within this article, facilitates two biochemical processes: (i) it acts as an antioxidant acting either directly on reactive oxygen species (ROS) or as a precursor to other antioxidants and (ii) it acts as a mucolytic compound that disrupts disulphide bonds in mucus glycoproteins. NAC has been used in other organoid models, such as intestinal and prostate organoid media (Aldini *et al.*, 2018), and was included in the media of both Turco *et al.* (2017) and Boretto *et al.* (2017); however, whether it is a necessary component has not been extensively explored. Nonetheless, assembloid co-culture models of endometrial glands and stroma seem to suggest an essential role of NAC in maintaining the epithelial phenotype in a simplified organoid medium (Rawlings *et al.*, 2021). Nicotinamide (NICO) is a form of vitamin B3 that is actively involved in nicotinamide adenine dinucleotide (NAD+) redox reactions involved in cellular homeostasis (Hwang and Song, 2017). In Turco *et al.* (2017), the removal of NICO caused the most significant reduction in size and number of EEOs across all media conditions tested, suggesting its importance to organoid growth. Lastly, insulin–transferrin–selenium (ITS) is a serum replacement also available commercially to promote cell growth at lower concentrations of FBS, or in the case of EEO media, the complete absence of FBS. ITS supports metabolic pathways through insulin and the mitigation of potential ROS through transferrin and selenium.

#### Antibiotics and antifungal agents

To prevent culture loss due to contamination, antibacterial, antifungal, or combination solutions are often used in primary cell culture, particularly following isolation from primary tissues. Penicillin/streptomycin (pen/strep) is a common antibiotic added to cultures at a concentration of 1% v/v. Established endometrial organoid media to date have used pen/strep or a commercial product named Primocin^®^ (InvivoGen, San Diego, CA, USA) to maintain healthy, uncontaminated cultures (Table 1). In one study investigating intestinal organoids, the growth and viability of primary cells were negatively impacted when exposed to pen/strep compared to Primocin^®^ during tissue washing steps (Marinucci *et al.*, 2022); however, this has not been reported in endometrial studies to our knowledge. Because organoids are derived from processed primary tissues, it is important to maintain careful aseptic technique when establishing cultures. Furthermore, performing mycoplasma testing for contaminants is highly recommended when establishing organoid cell banks.

#### Recombinant proteins and growth factors

To stimulate endometrial progenitor cells in the absence of serum and to mimic exogenous signals from the endometrial microenvironment, recombinant proteins are used to provide sufficient signalling factors and pathway activation, which sustains organoid cell proliferation and growth *in vitro*. Recombinant epidermal growth factor (rhEGF) is a common additive to human organoid cultures (Turco *et al.*, 2017). Regarding endometrial biology, rhEGF has been shown to influence the decidualization of endometrial stromal cells as a mediator of BMP2 and Wnt4 signalling (Large *et al.*, 2014). Due to the constant signalling between epithelial and stromal cells in the endometrium, growth factors secreted by the stroma are often included in EEO expansion media. These include the fibroblast growth factors, basic fibroblast growth factor (bFGF/FGF-2), or fibroblast growth factor 10 (FGF-10). *In vivo*, endometrial stromal cells are known to secrete FGF-10, which acts as a paracrine mediator for oestrogen-dependent proliferation in the uterus (Chung *et al.*, 2015). Hepatocyte growth factor (HGF), also produced by stromal endometrial cells (Sugawara *et al.*, 1997), supports the growth and proliferation of epithelial and stromal cells in the endometrium, and unregulated HGF/c-MET/Akt pathway activation is even shown to drive carcinogenesis in endometrial carcinoma (Li *et al.*, 2015). HGF has been included by some groups; however, its need in EEO organoid media appears to be species dependent as its inclusion did not have a significant impact on endometrial proliferation of mouse organoids (Boretto *et al.*, 2017), yet HGF withdrawal decreased the human EEO generation in two out of three donors in another study (Turco *et al.*, 2017). Several subsequent EEO protocols referenced in Table 1 do not use HGF; thus, including HGF in endometrial organoid media appears optional (Boretto *et al.*, 2017; Hernandez-Gordillo *et al.*, 2020). As previously mentioned, activation of canonical Wnt signalling has been established as fundamental for the initiation and maintenance of organoid growth in other mucosal tissues (Clevers *et al.*, 2014). Comprehensively, all EEO media use some form of proteins from the BMP–Noggin and Wnt/R-Spondin signalling pathways to maintain progenitor-fuelled organoid development of epithelial cell types (Sato *et al.*, 2009; Turco *et al.*, 2017). Both Noggin and R-Spondin are strictly required, as organoid growth is not possible without them; however, only mouse endometrial organoids require additional treatment with exogenous Wnt3A in cell culture media (Boretto *et al.*, 2017); human endometrial organoids do not. In the case of R-Spondin and Noggin, these proteins are delivered in the form of either (i) recombinant protein for human organoids (Turco *et al.*, 2017; Gnecco *et al.*, 2023) or (ii) conditioned media from Wnt3A and R-Spondin-producing L cells, a fibroblast cell line isolated from murine adipose tissue for mouse organoids (Boretto *et al.*, 2017).

#### Inhibitors

The purpose of inhibitor use in organoid media is two-fold: (i) to maintain the stem/progenitor cells in an undifferentiated state and (ii) block exogenous signals from the ECM-derived hydrogels used to culture EEOs (e.g. Matrigel), which may impact cell behaviour. Together, this cocktail of inhibitors coupled with the growth factors in organoid media are necessary for culture longevity and success; however, depending on the hypothesis or experimental design, the inclusion of certain pathway inhibitors might impact cell response and pathways of interest. For example, A83-01 is used widely as a transforming growth factor (TGF)-β receptor inhibitor to reduce differentiation, particularly in epithelial cells capable of entering epithelial-to-mesenchymal transition (EMT). Due to the presence of exogenous TGF-β in tumour-derived Matrigel, A83-01 is routinely used in organoid models and often needed to prevent culture interference via these external signals. However, TGF-β signalling also plays major roles in endometrial cell–cell communication underlying processes such as decidualization and apoptosis (Bruner *et al.*, 1995). Furthermore, TGF-β signalling is dysregulated in diseases such as endometriosis (Ruiz-Meana *et al.*, 1999; Ni and Li, 2017) and endometrial cancer (Kriseman *et al.*, 2019; Monsivais *et al.*, 2019). Thus, while the inclusion of an inhibitor such as A83-01 is important for preventing terminal differentiation of organoid populations to maintain progenitor populations, it may also interfere with studies focusing on TGF-β signalling. Further, the addition of pharmacologic TGF-β inhibitors or genetic silencing of TGF-β signalling pathways has been shown to impact the behaviour and phenotype of murine endometrial organoids *in vitro* (Kriseman *et al.*, 2023). Thus, the use of inhibitors must be employed thoughtfully while acknowledging the impact they might have on biological outcomes. Another common inhibitor, SB202190, is a small molecule p38 MAPK inhibitor used with A83-01 to increase longevity in intestinal and breast cancer organoids through inhibition of apoptosis (Sato *et al.*, 2011; Yu and Huang, 2020). In the endometrium, p38 MAPK phosphorylation is significantly increased through oestradiol signalling, and the ratio of phosphorylated/total p38 MAPK levels is shown to be significantly higher in the stratum functional layer compared to stratum basalis of the normal human endometrium across both the proliferative and secretory phases of the menstrual cycle (Seval *et al.*, 2006). SB202190 is used variably among the research groups referenced in this work; thus, it is again recommended to be utilized at the discretion of the researcher but not deemed necessary for establishing organoids. In fact, Sato *et al.* demonstrated that the addition of SB202190 in intestinal organoid media prevented differentiation of intestinal epithelial cells into mucus-producing goblet cells (Sato *et al.*, 2011), though the impact of SB202190 on endometrial differentiation has not been extensively explored. The last inhibitor used in EEO media is Y27632, an inhibitor of the Rho kinase/ROCK pathway used selectively to help recover dissociated organoid (Sato *et al.*, 2009, 2011) and stem cell cultures (Watanabe *et al.*, 2007; Harb *et al.*, 2008; Kim *et al.*, 2021). In EEOs, ROCK inhibitor (ROCKi) is temporarily added to the media during expansion and passaging but then removed for long-term maintenance. This inhibitor is crucial in the first 3–4 days of culture to increase epithelial cell viability but can be removed after cells have begun to divide, as stated in the EPHect-EM-Organoid SOP (Supplementary File S1). Recently, work from Shibata *et al.* (2024) made use of the inhibitors discussed above to create a stripped-down media containing only recombinant EGF, GSK-3 inhibitor CHIR99021, p38 inhibitor SB202190, and ROCK inhibitor Y27632. While the authors did not directly compare this stripped-down media to other foundational media recipes, such as those developed by Turco *et al.* (2017) and Boretto *et al.* (2017), single-cell RNA sequencing of EEO cultures demonstrated genomic signatures of differentiated epithelial populations, and EEOs cultured in this media demonstrated hormone responsiveness via protein secretion and hormone receptor changes in response to hormone stimulation (Shibata *et al.*, 2024). Thus, we acknowledge that the use of inhibitors in organoid media is application- and research-dependent, yet we propose the fewest use of inhibitors or growth factors may be beneficial for reducing costs and variability while increasing translational capacity.

#### Sex hormone stimulation

The endometrium is a sex-hormone-dependent tissue, and thus it is imperative to consider the use of steroid hormones in all endometrial experimental models. Moreover, dysregulation in oestrogen (E2) and progesterone (P4) signalling is a characteristic hallmark of endometriosis (Patel *et al.*, 2017). EEOs capture this hormone responsiveness by expressing both E2 and P4 hormone receptors (Turco *et al.*, 2017) and undergo hormone-mediated differentiation, thereby providing relevant models to study sex hormone signalling in health and disease. While cyclical changes of E2 and P4 occur across the menstrual cycle, E2 is present at some level throughout the cycle during the reproductive years (Reed and Carr, 2000). Given this, it is recommended to encourage the proliferation of EEOs via the supplementation of expansion media with E2. Garcia-Alonso *et al.* (2021) have demonstrated that EEOs cultured for 48 h without E2 still demonstrate proliferative gene signatures; however, whether sustained organoid culture in the absence of E2 impacts functionality and growth has yet to be deeply explored. In addition to driving proliferation, E2 signalling elicits differentiation within EEOs at a single-cell level, namely through the upregulation of PGR and biosignatures associated with pre-ciliated cells *in vitro* (Garcia-Alonso *et al.* 2021). Researchers interested in avoiding oestrogen signalling of EEOs while investigating the stem cell populations of the endometrium might benefit from omitting E2 from their media for this reason. Characterization of organoid response to E2 and P4 by several groups has consistently revealed robust responses to stimulation resulting in morphologic alterations (e.g. epithelial maturation, differentiation, pseudostratification and pinopode formation (Luddi *et al.*, 2020), biochemical changes, e.g. PAEP secretion (Luddi *et al.*, 2021; Simintiras *et al.*, 2021), and molecular pathway activation (Hewitt *et al.*, 2022) associated with P4 signalling compared to oestrogen-driven gene expression. To induce secretory and glandular phenotypes, a combination of E2 and P4 or synthetic progestins, such as medroxyprogesterone acetate (MPA), should be added to endometrial organoid media (Gnecco *et al.*, 2023). To drive progesterone-mediated responses, cyclic adenosine monophosphate (cAMP) is frequently added to the media to enhance progesterone signalling. In combination, adding cAMP to E2 + P4 (or MPA) treatments significantly reduced genes for proliferation and enhanced expression of P4 responsive genes within 4 days, indicating that stimulation with cAMP allows for experiments with shorter timelines compared to E2 + P4 only treatments (Fitzgerald *et al.*, 2019; Luddi *et al.*, 2020; Rawlings *et al.*, 2021; Hewitt *et al.*, 2022). Human chorionic gonadotropin (hCG), secreted by trophoblasts, and human prolactin (hPL), secreted by decidualized stromal cells, have also been used in organoid models to mimic differentiation cues in organoid models of early pregnancy phenotypes (Turco *et al.*, 2017). The use of cAMP, hCG, and hPL, in combination increased expression of PAEP, a positive marker for secretory cells, and decreased progenitor SOX9 expression, indicating a shift from a proliferative to more differentiated phenotype in endometrial organoids (Turco *et al.*, 2017). Thus, *in vitro* hormone signalling can produce a variety of phenotypes relevant to physiological questions spanning distinct phases of endometrial physiology. It is therefore recommended that the researcher considers the scientific question, study pathway, and culture timeframe to identify the appropriate hormones for their EEO model.

#### ECM and biomaterials

A critical element for the culture of organoids is a hydrogel matrix, typically derived from natural ECM proteins, that provides the scaffolding that enables them to grow in 3D. The gold standard matrix for almost all organoid models is a basement membrane (BM) extract hydrogel, called Matrigel (or Celltrex), that is derived from Engelbreth–Holm–Swarm mouse sarcoma cells. Matrigel is rich in laminin, collagen IV, and other ECM components (Passaniti *et al.*, 2022) and has been very effective as an *in vitro* 3D substrate for organoid systems due to its complex composition and gelation properties at 37°C (Rinehart *et al.*, 1988; Boretto *et al.*, 2017; Turco *et al.*, 2017). However, due to its animal tumour origin and highly heterogeneous composition, Matrigel confers numerous limitations that restrict certain applications for mechanistic and translational studies. Given that Matrigel is sourced from a mouse sarcoma cell line, xenogeneic contamination is a concern when working with human-sourced cells, eliminating any options for clinical applications in regenerative medicine (Aisenbrey and Murphy, 2020; Fogg *et al.*, 2023). Additionally, the precise composition of Matrigel (i.e. protein concentrations and presence of endogenous cytokines or growth factors) is highly undefined and exhibits lot-to-lot variability, thereby limiting its biomechanical tunability and prohibiting the incorporation of additional non-epithelial cell populations, such as stromal or immune cells. Given these drawbacks, recent efforts have been made to use alternative hydrogels for the culture of EEOs that overcome these shortcomings by recapitulating the native composition and biophysical properties of the endometrial ECM (EndoECM). Approaches to generate hydrogel alternatives to Matrigel for endometrial organoids have led to two main strategies: (i) top-down approaches using the native EndoECM proteins as a hydrogel biomaterial and (ii) bottom-up approaches that use synthetic building blocks or naturally derived ECM proteins as biomaterials to incorporate a minimal set of parameters necessary to maintain organoid culture and endometrial physiology for *in vitro* applications.

##### Alternative biomaterials: collagen-derived ECM hydrogels

Bottom-up approaches rely on naturally derived or recombinant ECM proteins, such as fibrillar collagens and fibronectin, as hydrogels to support endometrial cell growth. Collagen I hydrogels are the most common biologically sourced alternatives to Matrigel when culturing endometrial cells (Kirk and Alvarez, 1986), as their mechanical tunability, affordability, and defined composition enable numerous *in vitro* applications. Abbas *et al.* (2020) used type I bovine collagen through a process of freezing and lyophilization to produce 3D scaffold sponges with a mesh size of ∼100 µm, allowing for endometrial cell infiltration and growth. These collagen scaffolds supported successful epithelial differentiation, lumen formation, and stromal decidualization response (Abbas *et al.*, 2020). While this model did not directly use EEOs in the hydrogel, it set a strong precedent for modelling multicellular cultures by mimicking tissue architecture and providing greater control over material tunability. Recently, Rawlings *et al.* (2021) generated a co-culture model of EEOs and stromal fibroblasts using a 97% collagen I, 3% collagen III composite that mimics the dominant collagen proteins in the endometrium. This platform enabled the generation of one of the first organoid models to examine early events in blastocyst–endometrial interactions, highlighting the role of stromal subpopulations in implantation (Rawlings *et al.*, 2021). Unfortunately, due to the fast consumption rate of collagen by stromal and epithelial cells, collagen hydrogels are limited to constrained short time-scale experiments (4–6 days) before the gels start to fall apart. Finally, although the biomechanical properties of these collagen gels can be partially adjusted by increasing collagen protein concentration, crosslinking, and fibre organization, these mechanical adjustments are limited and do not fully mimic native tissue regimes (Sarrigiannidis *et al.*, 2021).

##### Alternative biomaterials: decellularized tissue ECM hydrogels

A top-down biomaterial approach aims to capture all the components of the tissue matrisome and leverages tissue decellularization techniques to use native endometrium ECM to make a hydrogel (Francés-Herrero *et al.*, 2021; López-Martínez *et al.*, 2021; Jamaluddin *et al.*, 2022). Tissue decellularization involves using a series of detergent washes to remove cellular material from intact tissue, leaving an acellular scaffold matrix that can be used to study the matrisome, reseeded with cells, or further processed to create a hydrogel substrate. In both cases, utilizing tissue decellularization allows the researcher to recapitulate a tissue’s native extracellular matrix. Tissue decellularized substrates have been used to culture EEOs and have demonstrated improved proliferation of endometrial stem cells compared to those grown in Matrigel or collagen. Furthermore, this decellularized ECM hydrogel can further support the simultaneous culture of endometrial epithelial and stromal cells in transwell assay cultures (López-Martínez *et al.*, 2021). Collectively, these studies show that using extracts from EndoECM as an exogenous biomaterial can enhance organoid cultures and have strong implications for regenerative medicine. To support this, a follow-up study further explored the use of decellularized porcine EndoECM as a soluble additive to improve organoid growth conditions (Francés-Herrero *et al.*, 2021). Another group deployed a similar decellularization approach that utilized a hydrogel derived from bovine and human EndoECM to support organoid growth of healthy and cancerous endometrial cells (Jamaluddin *et al.*, 2022). This ECM hydrogel approach enabled the generation of EEOs with comparable efficiency to Matrigel in both bovine- and human-derived hydrogels (Jamaluddin *et al.*, 2022). The main advantage to these biomaterial approaches is the ability to capture ECM complexity in a hydrogel that may help elucidate the role of matrisome dysregulation in endometriosis. However, the use of decellularized ECM extracts still presents challenges due to its variability and undefined substrate composition. As previously discussed, extraneous pathway growth factors and cytokines present in naturally derived and undefined ECM extracts might unknowingly interfere with specific pathways intended for study or prove difficult to replicate if the composition of the hydrogel is poorly defined.

##### Alternative biomaterials: synthetic ECM hydrogels

Compared to naturally derived ECMs, synthetic matrices are rationally designed to mimic the parameters of tissue ECM that enable the growth of organoids in defined and controlled conditions. Numerous approaches for engineered hydrogels have been developed for organoids of many tissues, including the endometrium (Gjorevski *et al.*, 2016; Aisenbrey and Murphy, 2020; Hernandez-Gordillo *et al.*, 2020; Gnecco *et al.*, 2023). Fully synthetic and semi-synthetic composite biomaterials have recently been proposed by Blatchley and Anseth (2023) as the ‘middle-out’ solution to hydrogel design. With greater control over matrix properties of the gels, such as the mechanical properties, integrin-binding peptide availability, and enzyme-digestible crosslinkers, researchers now have the capability to provide supportive growth environments without the interference of undefined exogenous factors found in naturally derived hydrogels such as Matrigel or decellularized endometrium. An advantage of this middle-out approach is the ability to tailor suitable environments for specific tissues of interest by providing the necessary cues to sustain cell growth and differentiation (Lutolf and Hubbell, 2003; Griffith and Swartz, 2006). For endometrial organoids, synthetic hydrogels have been developed for both EEOs and multicellular cultures (Cook *et al.*, 2017; Hernandez-Gordillo *et al.*, 2020; Gnecco *et al.*, 2023). These matrices are comprised of a bioinert polyethylene glycol (PEG)-based macromer backbone that is functionalized with specific cell adhesion ligand peptides, such as PHSRN-K-RGD and GFOGER, that engage the integrins expressed by cells that bind to fibronectin and collagen, respectively. Additionally, these PEG hydrogels are functionalized with ECM-binding domains specifically designed for sequestering BM and fibronectin proteins produced by the cells, allowing embedded cells to influence the ECM environment. Finally, MMP-labile crosslinkers are used to polymerize the hydrogel and enable cell-initiated remodelling of the synthetic ECM (Cook *et al.*, 2017; Valdez *et al.*, 2017; Hernandez-Gordillo *et al.*, 2020; Gnecco *et al.*, 2023). Altogether, by functionalizing with multiple binding domains, PEG gel formulations have been identified that allow for the generation, culture, and maintenance of endometrial organoids with >75% efficiency for long-term (>14 days) cultures (Hernandez-Gordillo *et al.*, 2020; Gnecco *et al.*, 2023). Furthermore, these hydrogels are capable of supporting non-epithelial cells as well due to the incorporation of dual-adhesion ligands that can engage mesenchymal populations (Gnecco *et al.*, 2023). These synthetic hydrogels also enable greater control of the bulk biophysical properties of the matrices, thereby enabling studies of the cell–ECM interactions across both healthy and diseased regimes (Gnecco *et al.*, 2023). Synthetic matrices further offer the benefit of sustained mechanical integrity for prolonged culture periods compared to other natural hydrogels mentioned herein (Rawlings *et al.*, 2021). Finally, because synthetic biomaterials are fully defined and animal-free and enable the generation of organoids without the need to ever expand in Matrigel, they enable opportunities for clinical translation. In sum, it is important to recognize the types of cells, media, and biomaterial tools available for organoid research, such that they can be acutely adapted for hypothesis testing and used for studying endometriosis.

### Implementing organoids for reproductive disease modelling

**Figure 3.**
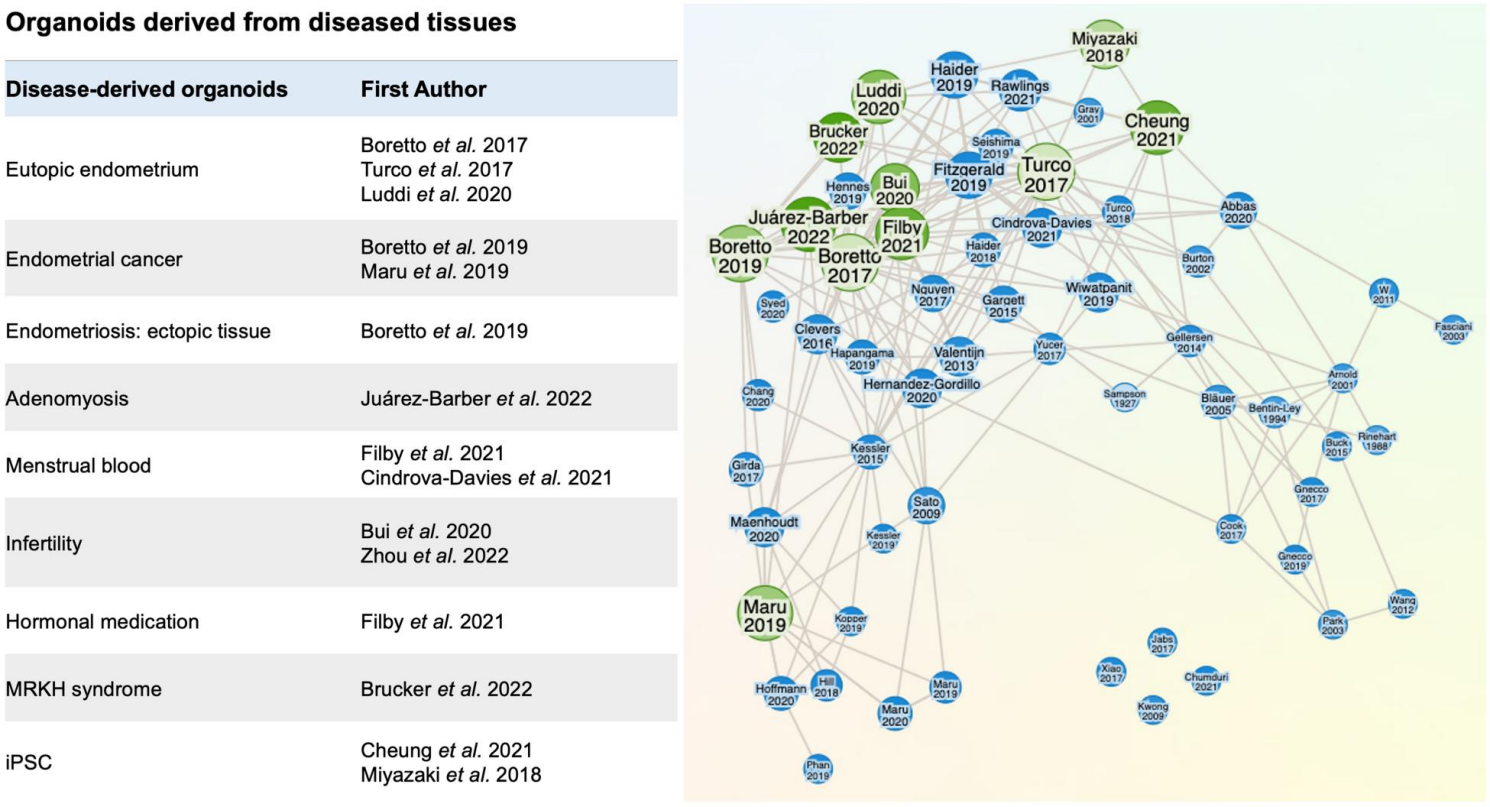
**Disease modelling of endometrial disorders using organoids.** Multiple studies have generated endometrial organoids from diseased or unique tissue sources, as highlighted in the leftmost panel. Research Rabbit was utilized, an open-source platform that collates relevant papers based on a cited collection (citations from the leftmost list were input and are highlighted in green), which connects researchers to associated works based on citations and manuscript tags (displayed in blue). This platform was utilized to ensure comprehensive coverage of the organoid field, and many highlighted blue citations are discussed within this article.

Current EEO research is primarily derived from the eutopic endometrial tissues of endometrial donors and also from animal models, including mice and some domestic animals (Boretto *et al.*, 2017; Kruitwagen *et al.*, 2017; Thompson *et al.*, 2022b). However, the generation of EEOs from clinically diagnosed patient populations suffering from gynaecological pathologies presents a particularly exciting opportunity to advance the number and quality of experimental models of endometrial disease. To date, EEO lines have been generated from endometrial biopsies of patients with several gynaecological disorders, including endometriosis (Filby *et al.*, 2021; Gnecco *et al.*, 2023), adenomyosis (Juárez-Barber *et al.*, 2022), low-to-high grade endometrial cancer (Maru *et al.*, 2019; Boretto *et al.*, 2019), Mayer–Rokitansky–Küster–Hauser (MRKH) syndrome (Brucker *et al.*, 2022), and infertility (Bui *et al.*, 2020; Zhou *et al.*, 2022). These patient-derived organoids serve as novel *in vitro* research tools for capturing the phenotypic and genetic landscape of these diseases (Fig. 3). For example, EEOs have recently been used to evaluate changes in epigenetic HOX gene methylation at specific sites in endometriosis patients, showing that these organoids retained the genomic profile of the original disease *in vitro* (Esfandiari *et al.*, 2021). In other studies, EEOs helped confirm that these patients sustained dysregulated sex hormone signalling, including the decreased expression of PAEP, a glycodelin secreted by the epithelium in response to progesterone, by comparing healthy and diseased endometria (Brucker *et al.*, 2022; Hewitt *et al.*, 2022). Similarly, studies using EEOs from patients with infertility showed that these patients have reduced expression of receptivity markers, including hydroxysteroid 17-beta dehydrogenase 2 (HSD17B2), FOXO1, and MUC1 in response to treatment with oestradiol, progestin, and cAMP, suggesting a dysregulated response in hormone-responsive genes (Zhou *et al.*, 2022). Complementary studies analysed the intra-organoid-fluid (IOF) from fertility, and infertile patient organoids further revealed that MUC5AC was significantly increased in infertile IOF compared to fertile IOF, a finding corroborated via proteomic analysis (Zhou *et al.*, 2022). Altogether, these early studies demonstrate the potential of EEOs for modelling disease *in vitro* and their utility for studying endometrial disease, particularly for endometriosis.

Organoids will provide a major benefit for endometriosis research due to their ability to be generated from both eutopic and ectopic lesion tissues (Koninckx *et al.*, 2021; Esfandiari *et al.*, 2022). Boretto *et al.* (2019) were the first to report the generation of organoids from endometriotic ectopic lesions. Lesions derived EEOs (LEEOs, to be consistent with the EEO terminology used in this review) can be generated from ectopic tissues with relatively high efficiency using protocols similar to those used for eutopic endometrial organoids. Due to the clonal properties of organoid culture, this enables their expansion and biobanking. Thus, even with a small lesion sample, only a few cells are needed to establish the initial colony of LEEOs, which can then be expanded and characterized to generate a living model of endometriotic epithelial disease *in vitro* (unpublished data). Growing donor-matched organoids from eutopic (EEOs) and ectopic disease (LEEOs) is highly encouraged whenever possible, as it serves as an ideal internal control for diseased patients to assist in the interpretation of patient heterogeneity. As more LEEO lines from distinct endometriotic lesions are generated and become established, it will be important for the endometrial organoid research community to define validation metrics to ensure clinical relevance, purity, and endometriotic disease phenotype, especially when comparing lesion subtypes. This is an area of research that remains to be defined; however, a recommended minimal panel could include epithelial morphologic assessment (lumenized epithelial structures >100 µm), expression or lack thereof of hormone receptors (e.g. EpCAM+, ESR-1/2, and PGR-A/B receptors), as well as a clinical histological correlate and a comprehensive clinical record of disease stage and location as a suitable starting point. An idealized pipeline to establish LEEOs would also include clearly documented lesion metadata, including histological characterization of the eutopic tissue for endometrial cycle dating. Altogether, disease organoid models will represent a functional and clinically relevant *in vitro* model of disease that, when coupled to analytical tools and multi-omic approaches, including genetic (e.g. exome sequencing (Boretto *et al.*, 2019)), transcriptomic (bulk and single-cell RNAseq (Turco *et al.*, 2017; Vento-Tormo *et al.*, 2018; Fitzgerald *et al.*, 2019; Garcia-Alonso *et al.*, 2021; Zhang *et al.*, 2024)), proteomic (Simintiras *et al.*, 2021; Thompson *et al.*, 2022a), and metabolic analysis, will advance our understanding and treatment of endometriotic diseases.

### Limitations and challenges of organoid technologies

Despite the unprecedented benefits of organoids for *in vitro* modelling of healthy and diseased endometrium, their limitations must be recognized to adequately apply and interpret EEO-mediated research and to design appropriate experiments based on the research questions (Supplementary File S1). First, organoid models can be cost-prohibitive due to the highly customized media requiring numerous recombinant growth factors and inhibitors, in addition to the hydrogel (e.g. Matrigel) material costs; the volumes and frequent use of these materials are further described in the EPHect-EM-Organoid SOP (Supplementary File S1). Secondly, accessibility to clinical primary tissues may be restricting for some groups and will require technical expertise in tissue isolation protocols. Moreover, a lack of standardization for clinical phenotyping and validation protocols for disease-derived organoids may hinder the interpretation of data generated using these models. Thus, the establishment of accessible endometriosis organoid biobanks and the standardization of universal organoid validation/characterization pipelines, similar to what has been done previously in other consortia for endometrial primary tissues (Romano *et al.*, 2022) and cancer research (Adishesh *et al.*, 2017), are needed.

There remain research areas where EEOs are not suitable models due to their technical limitations and oversimplification. For example, access to the apical side of EEOs remains a major challenge. The apical–basolateral polarity of epithelial glands is important to maintain tissue function, and differences in secreted factors between these two compartments have been shown in EEOs (Simintiras *et al.*, 2021; Zhou *et al.*, 2022). However, the generation of stratified components that capture both the contiguous glandular and luminal components of endometrial epithelia remains difficult. Future development of organoid systems that enable access to the apical side and capture the luminal–glandular epithelial heterogeneity will help advance our understanding of blastocyst implantation, infection, barrier function, or epithelial apical–basolateral function. Another limitation in EEOs models results from a lack of other cellular components (e.g. stromal, immune, or vascular elements) and the associated signalling networks observed in the tissue microenvironment *in vivo*. For example, while EEOs reproduce some key facets of sex hormone signalling, analysis of the genomic cistrome in response to P4 stimulation shows that they do not fully mimic those of the intact tissue (Hewitt *et al.*, 2022). This observation could be due to a combined lack of the stromal populations in the system and premature protein priming by the presence of P4 in the organoid media supplements. While EEOs are a powerful tool for studying the epithelium of the endometrium, these are areas that still require further development. Increased sophistication of models and emerging ‘multi-cellular organoid systems’, also termed ‘assembloids’ (Rawlings *et al.*, 2021), may help address these needs.

### Next-generation *in vitro* organoid systems: modelling cellular communication in endometriosis

**Figure 4.**
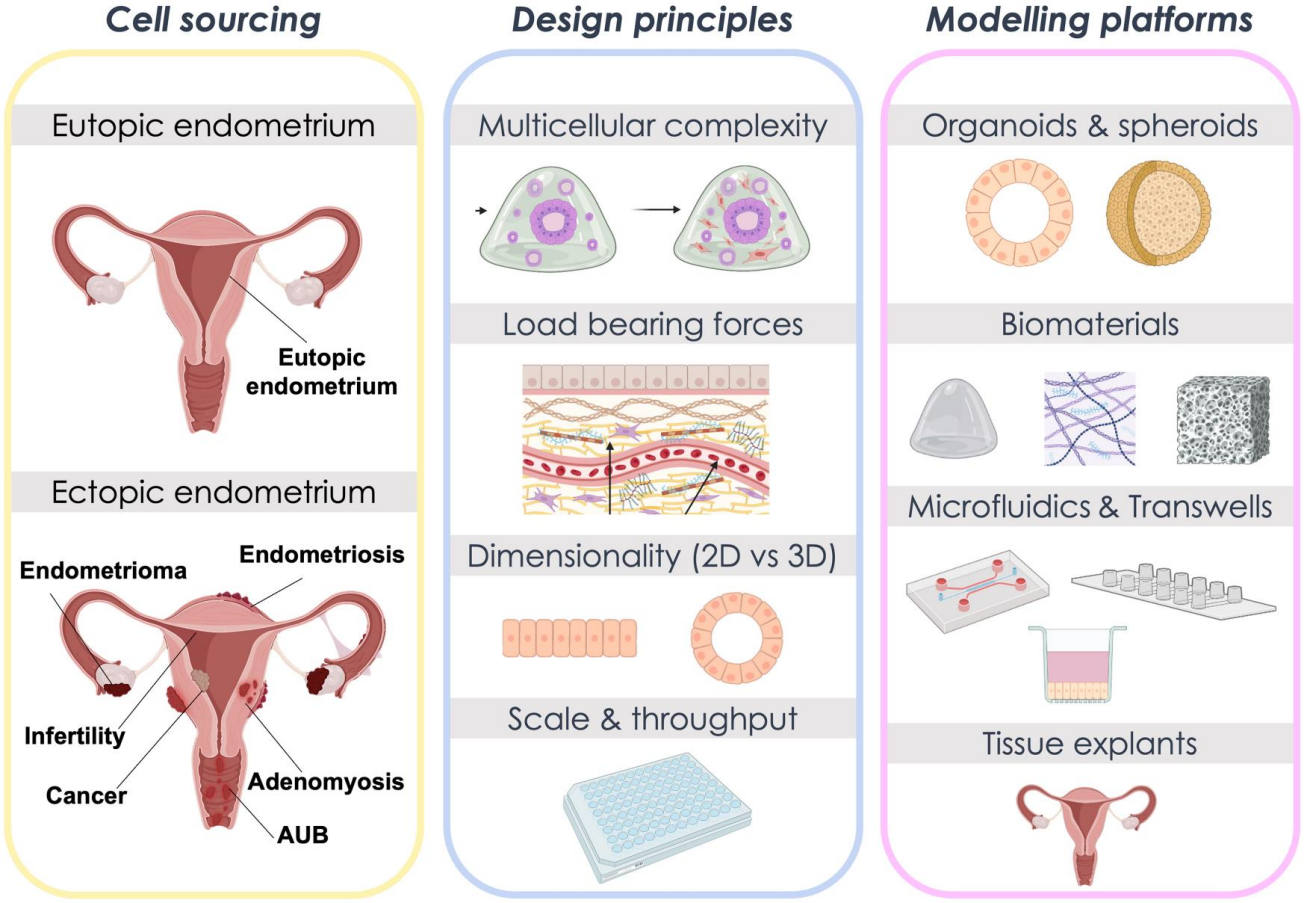
**Existing *in vitro* technologies for endometriosis research.** An overview of the range of desired applications, design principles to consider, and existing cell sources and modelling platforms that can be used to perform *in vitro* endometrial research. Cell sourcing from healthy or diseased tissues can be paired with design principles that mimic the *in vivo* environment pertinent to the research question at the appropriate scale and throughput. These design principles include the mechanics, dimensionality, cellular complexity, and throughput of systems to yield different modelling platforms for healthy or diseased endometrial *in vitro* models. Far left panel: AUB, abnormal uterine bleeding. Created in BioRender. Marr (2025). https://BioRender.com/caj893u.

Innovations in biomaterials and *in vitro* platforms are enabling more physiologic and complex organoid models. *In vivo* and murine renal graft models using recombinant tissues from genetically modified animals have demonstrated that stromal–epithelial crosstalk is fundamental to maintaining physiologic endometrial reproductive function (Cooke *et al.*, 1998). To explore this concept *in vitro*, there is strong interest in using organoids to build multicellular models that incorporate additional cellular components of the tissue-level microenvironment (Fig. 4). For example, advances in biomaterials and microphysiological systems (MPS) have helped establish stable co-culture models of EEOs and endometrial stromal cells (ESCs), a feat that is prohibitive in Matrigel due to its exogenous signalling, heterogeneous composition, and incompatibility with mesenchymal cells (Gnecco *et al.*, 2023). These approaches, referred to as ‘assembloids’ (Rawlings *et al.*, 2021), ‘multicellular organoids’ (Wiwatpanit *et al.*, 2020; Song and Fazleabas, 2021), and ‘organoid co-cultures’ (Cheung *et al.*, 2021; Gnecco *et al.*, 2023), comprise the broad class of ‘organoid systems’, a term that captures the next-generation organoid-derived multicellular cultures and reflects the systems-level multi-omic approaches used to analyse these models.

To date, organoid-based co-culture models have been developed using both hydrogels and scaffold-free approaches to simultaneously culture and study stromal–epithelial interactions in endometriosis research (Fig. 4). These co-culture models have been used to assess the trajectory of stromal–epithelial subpopulations in response to hormone stimulation and have been leveraged to model the process of blastocyst implantation *in vitro* (Rawlings *et al.*, 2021). Another example of an organoid–stromal co-culture was developed using a synthetic PEG hydrogel and enabled the long-term simultaneous culture of these two populations *in vitro* that functionally captured key hormone-mediated cellular and molecular changes associated with the distinct phases of the menstrual cycle (Gnecco *et al.*, 2023). This co-culture model revealed the impact of epithelial–stromal communication in response to progestin signalling and furthermore demonstrated that the pro-inflammatory cytokine IL-1β signalling impacted epithelial proliferation via an indirect mechanism mediated through the stroma (Gnecco *et al.*, 2023). Early evidence from these multicellular organoid systems points to the importance of incorporating additional cell types into *in vitro* models of endometrial dysfunction. Work using rodent endometrial co-cultures with peritoneal cells has demonstrated that paracrine communication can influence the proliferative behaviour of endometriotic cells, and further human co-culture studies show that the altered progesterone signalling and MMP expression observed in the endometriotic phenotype are mediated by dysregulated stromal–epithelial communication (Sharpe *et al.*, 1992; Igarashi *et al.*, 2005). These early studies highlight the importance of incorporating and dissecting the underlying cellular communication in endometriosis. To emulate the lesion microenvironment, future directions should continue to explore not only epithelial–stromal crosstalk but also other cell populations of the endometrial lesion microenvironment, including immune cells, endothelial cells, or cells derived from the peritoneal mesothelium.

Alternative examples for modelling cellular crosstalk *in vitro* are scaffold-free spheroids, a 3D multi-cellular model, whereby, in comparison to EEOs, aggregates of stromal and epithelial populations form spherical tissue constructs (Wiwatpanit *et al.*, 2020). Generation of spheroids can be performed using low-adhesion culture plates, bio-fabricated organotypic tissue constructs (Wendel *et al.*, 2020), or micro-moulded agarose wells (Wiwatpanit *et al.*, 2020), or by hanging drop methods (Stejskalová *et al.*, 2021). While spheroids are more reproducible and scalable than organoids due to the reduced dependency on a hydrogel scaffold, the lack of an epithelial lumen often leads to the development of a necrotic core, and thus spheroids are prone to developing apoptotic bodies resulting from a lack of oxygen and nutrient diffusion within these constructs (Thompson *et al.*, 2022a). Nonetheless, these models have been useful in advancing endometriosis research by helping reveal that the ECM microenvironment is a factor that contributes to endometriosis invasion (Stejskalová *et al.*, 2021). Spheroid studies have also been useful in mechanistic and preclinical pathways, including the NOTCH pathways (Strauß *et al.*, 2022) in endometriosis pathogenesis. Although many of these spheroid models have primarily relied on the endometriotic cell lines (e.g. 12Z), future approaches, using primary tissue-derived EEOs to make spheroids, may provide greater translational potential to model contact-based cell–cell communication in endometriosis (Murphy *et al.*, 2019).

**Figure 5.**
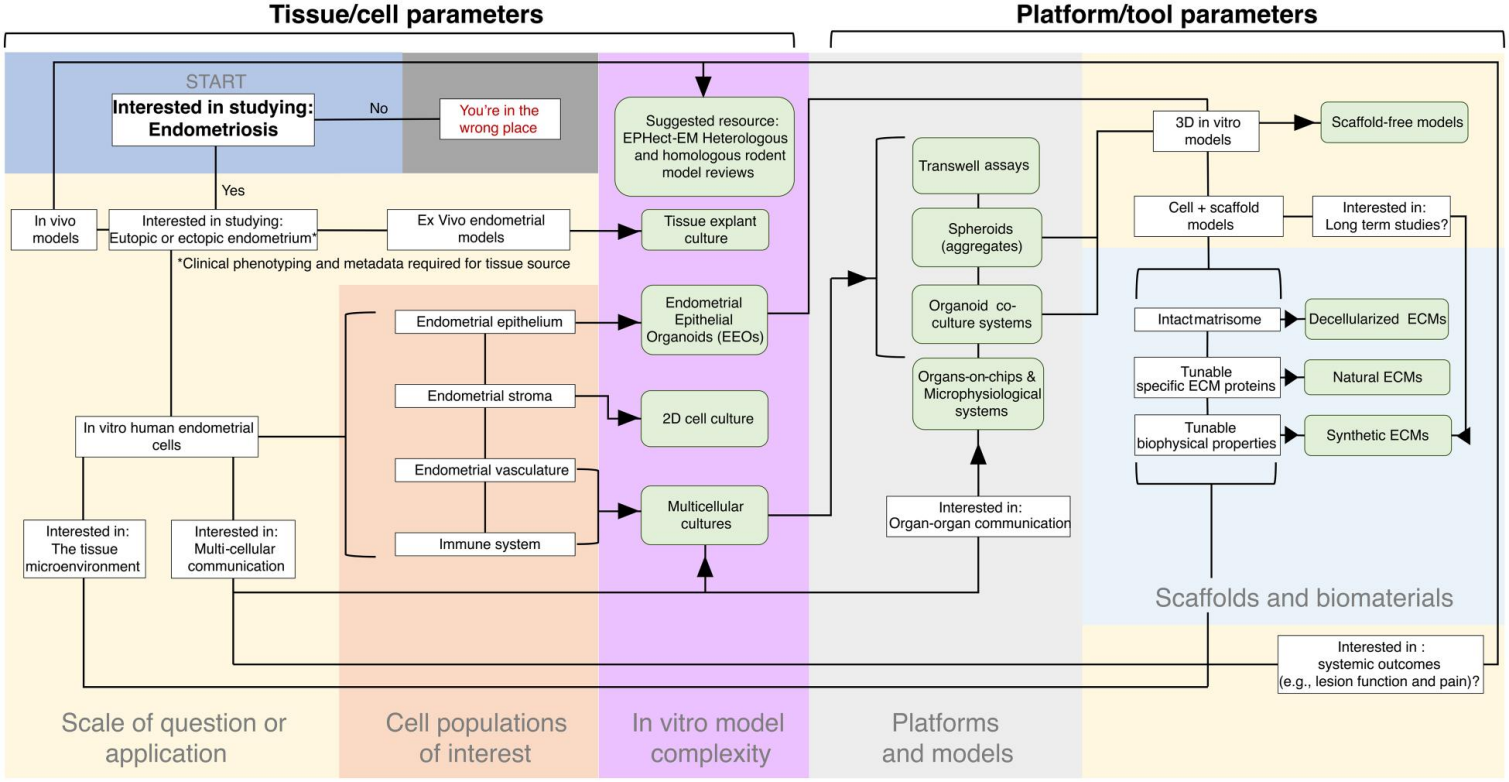
**A decision tree framework for guiding endometriosis model selection.** Beginning at the top left, responses will guide researchers to models best suited for their experimental set-up, and are focused on cell populations of interest, scale, microenvironment, and disease state. This decision tree is a starting framework focused on *in vitro* models such as those covered in this paper. ECM, extracellular matrix.

Finally, physiomimetic modelling of both the cellular and biophysical composition of the endometriotic lesion microenvironment is currently being developed (as reviewed in detail elsewhere (Grümmer, 2012; Deane *et al.*, 2017; Fan, 2020; Gnecco *et al.*, 2020; Hibaoui and Feki, 2020)). Recent developments in MPS model systems includes heterotypic multicellular models that recapitulate features of the microenvironment, such as tissue-level architecture (Fig. 4). For example, increased model complexity as observed with sophisticated MPS platforms, including organ-on-chip models, enables the modelling of tissue-level processes, such as incorporation of immune or vascular populations, and allows for investigation into the role of physiological conditions (e.g. haemodynamic forces) or biophysical properties (e.g. stiffness) on cellular behaviour (Gnecco *et al.*, 2019). The merging of these prototypical models and EEOs will inherently provide more physiological and clinically relevant approaches for modelling disease. It is important to recognize that the selection of the model should ideally be driven by a defined hypothesis-driven question, which will guide which model is needed. There is no one model system that will be sufficiently complex and comprehensive to address all the parameters of endometriotic disease; therefore, the model must be designed or chosen with the goal of addressing specific study objectives or testing a hypothesis. Every model is limited by the scalability (i.e. the assay throughput) and its complexity, which are often inversely correlated. We provide an initial framework to help guide selection of the types of *in vitro* models for specific research questions (Fig. 5). Finally, we propose that the use of *in vitro* models for endometriosis research should also be accompanied by specific output metrics and an appropriate analytical plan (e.g. transcriptomic, proteomic, and imaging approaches) to fully leverage the mechanistic capability of these *in vitro* models.

## Discussion

Endometriosis remains one of the most poorly understood conditions due to its molecular, hormonal, cellular, and heterogeneric complexity, and an overall lack of funding for research into female-only diseases (Smith, 2023; Zondervan *et al.*, 2023). Thus, organoids are a promising innovative tool to investigate the mechanistic features of endometriotic aetiology and advance the identification of potential novel therapeutic targets to treat those whose lives are compromised by endometriosis. We recognize that EEOs are an emerging technology and an experimental model that presents both opportunities and limitations for *in vitro* modelling of endometriosis. It is, therefore, important to unify the protocols and standard operating procedures (SOPs) for generating, using, and analysing organoid-derived models (Supplementary File S1). Such efforts to improve current technical methodologies will increase translational potential and overcome limitations that currently hinder reproducibility within a single laboratory and between different research groups. Additionally, it is critical to increase accessibility to these model systems in the field. To do so, method unification, transparency, biobanking, and robust clinical phenotyping of patient-derived samples will be necessary to fully leverage organoids as a powerful resource for advancing the gynaecological field and reproductive biology. Specifically for endometriosis, organoids offer an unprecedented opportunity to study this complex disease by using ectopic tissue and enabling the generation of LEEOs, a living model of endometriotic epithelium. Thus, a comprehensive panel of specific hallmarks of endometriosis, including, but not limited to, validation of cancer-associated mutations, progesterone resistance, oestrogen dependency, and invasive behaviour, is needed to use organoids accurately and appropriately for endometriosis research. Coupling organoids with *ex vivo* and *in vivo* approaches, such as experimental animal models and *ex vivo* analysis of clinical samples whenever possible, is essential to advance translational relevancy. As emerging endometrial organoid system models continue to be developed, we can expect a deeper mechanistic understanding of the aetiology of endometriosis and the identification of novel therapeutic targets to benefit the millions whose lives are compromised daily due to endometriosis.

## Supplementary Material

gaaf024_Supplementary_Data

## Data Availability

No new data were generated or analysed in support of this research.

## Appendix: EPHect Experimental Models Working Group

Nick A. Andrews: Salk Institute for Biological Sciences, San Diego, CA, USA

Michael S. Anglesio: University of British Columbia, Vancouver, BC, Canada

Caroline B. Appleyard: Ponce Health Sciences University-Ponce Research Institute, Ponce, PR, USA

Joe Arosh: Texas A&M University, College Station, TX, USA

Christian M. Becker: University of Oxford, Oxford, UK

Kaylon L. Bruner-Tran: Vanderbilt University Medical Center, Nashville, TN, USA; World Endometriosis Research Foundation, London, UK

Katherine A. Burns: University of Cincinnati College of Medicine, Cincinnati, OH, USA

Ronald L. Chandler; Michigan State University College of Human Medicine, Grand Rapids, MI, USA

Julie A. Christianson: University of Kansas Medical Center, Kansas City, MO, USA

Fiona L. Cousins: Hudson Institute, Clayton, VIC, Australia; Obstetrics and Gynaecology, Monash University, Clayton, VIC, Australia

Kelsi N. Dodds: Flinders University, Adelaide, SA, Australia; University of Adelaide, Adelaide, SA, Australia

Victor Fattori: Harvard University, Cambridge, MA, USA

Asgi Fazleabas: Michigan State University, Grand Rapids, MI, USA

Caroline Gargett: Hudson Institute of Medical Research, Clayton, VIC, Australia

Juan S. Gnecco: Tufts University, Medford, MA, USA

Raul Gomez; INCLIVA Instituto de Investigación Sanitaria, Valencia, Spain; University of Valencia, Valencia, Spain

Martin Götte: University of Münster, Münster, Germany

Erin Greaves: University of Warwick, Warwick, UK; World Endometriosis Research Foundation, London, UK

Linda G. Griffith: Massachusetts’s Institute of Technology, Cambridge, MA, USA

Patrick G. Groothuis: Byondis BV, Nijmegen, The Netherlands

Ruth Grümmer: University of Duisburg-Essen, Essen, Germany

Sun-Wei Guo: Fudan University, Shanghai, China

Shannon M. Hawkins: Indiana University School of Medicine, Indianapolis, IN, USA

M. Louise Hull: The Robinson Research Institute, University of Adelaide, Adelaide, SA, Australia

Lone Hummelshoj: World Endometriosis Research Foundation, London, UK

Mark R. Hutchinson: University of Adelaide, Adelaide, SA, Australia

Mohamed Gamal Ibrahim: Team Kinderwunsch Oldenburg GbR MVZ, Oldenburg, Germany

Elizabeth E. Marr: Tufts University, Medford, MA, USA

Stacy L. McAllister: Emory University School of Medicine, Atlanta, GA, USA

Stacey A. Missmer: Harvard TH Chan School of Public Health, Boston, MA, USA; University of Michigan, Ann Arbour, MI, USA; World Endometriosis Research Foundation, London, UK

Jeffrey S. Mogil: McGill University, Montreal, QC, Canada

Jens Nagel: Merz Therapeutics, Frankfurt, Germany

Warren B. Nothnick: Kansas University Medical Center, Kansas City, MO, USA

Paulina Nunez-Badinez: Bayer AG, Wuppertal, Germany (until 23 March 2023)

Kevin G. Osteen: Vanderbilty University Medical Center, Nashville, TN, USA

Daniëlle Peterse: Boston Childrens Hospital, Harvard Medical School, Boston, MA, USA

Michael S. Rogers: Boston Childrens Hospital, Harvard Medical School, Boston, MA, USA

Andrea Romano: Maastricht University, Maastricht, The Netherlands

Philippa T.K. Saunders: University of Edinburgh, Edinburgh, UK

Miguel Ángel Tejada: University of Granada, Granada, Spain

Kathy L. Sharpe-Timms: University of Missouri School of Medicine, Columbia, MO, USA

Waldiceu A. Verri: State University of Londrina, Londrina, Brazil

Paola Viganó: Fondazione IRCCS Ca’ Granda Ospedale Maggiore Policlinico, Milano, Italy

Katy Vincent: University of Oxford, Oxford, UK
