# Supplementary material for: WERF Endometriosis Phenome and Biobanking Harmonisation Project for Experimental Models in Endometriosis Research (EPHect-EM-Organoids): endometrial organoids as an emerging technology for endometriosis research
Source: Mol Hum Reprod. 2025 Jul 9;31(3):gaaf024. doi: 10.1093/molehr/gaaf024 (PMC12237518; doi:10.1093/molehr/gaaf024)
Supplement: gaaf024_Supplementary_Data [file gaaf024_supplementary_data.zip › MHR-24-0373-R1_Supplementary info.pdf]

**Supplementary Information**

# World Endometriosis Research Foundation EPHect Experimental Models for Endometriosis Research (EPHect-EM-Organoids): endometrial organoids as an emerging technology for endometriosis research

Elizabeth E. Marr, Juan S. Gnecco, Stacey A. Missmer, Shannon M. Hawkins, Kevin G. Osteen, Lone Hummelshoj, Erin Greaves, and Kaylon L. Bruner-Tran, for the EPHect-Experimental Models Working Group

Supplementary Figure S1: Timeline and framework of EPHect initiative

Supplementary Table S1: Utilized working concentrations and suppliers of media components from cited work discussed in Table 1

Supplementary File S1 EPHect Standard Operating Procedure Experimental models: organoids (provided as a separate file)

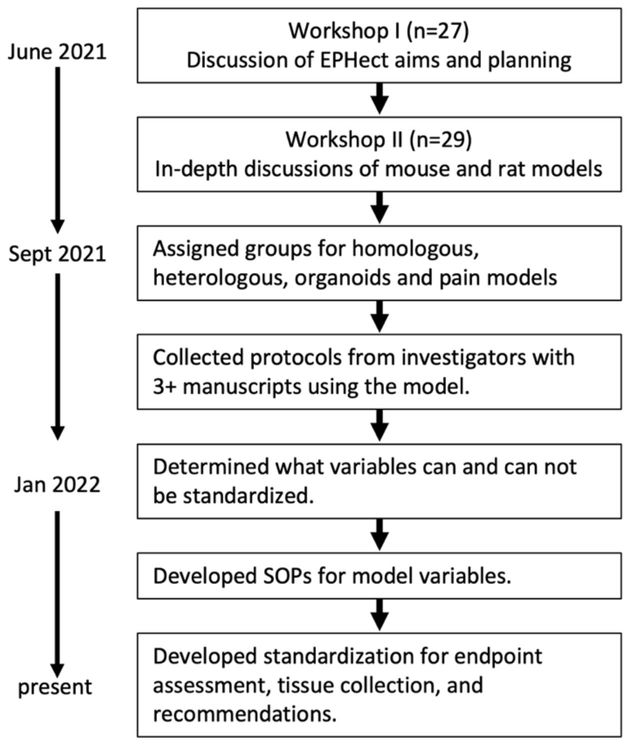


**Supplementary Figure S1: Timeline and framework of EPHect initiative.**

**Supplementary Table S1: Utilized working concentrations and suppliers of media components from cited work discussed in Table 1.**

| Category | Reagent | Primary use/notes | Working Concentration | Supplier |
|---|---|---|---|---|
| *Base media* | Advanced DMEM/F12 | Not available without phenol red | NA | ThermoFisher |
| | DMEM/F12 | With or without phenol red | NA | ThermoFisher |
| *Conditioned media* | R-spondin-1 media | Used in place of recombinant R-spondin | 10-25% | Contact authors |
| | Wnt3A media | Used in place of recombinant Wnt3A | 25% | Contact authors |
| *Recombinant protein* | Recombinant human EGF | Epidermal Growth Factor | 50 ng/mL | Peprotech, R&D Systems |
| | Recombinant human bFGF/FGF-2 | Fibroblast Growth Factor-2, also known as FGFbasic | 2 ng/mL | R&D Systems |
| | Recombinant human FGF-10 | Fibroblast Growth Factor-10 | 50-100ng/mL | Peprotech |
| | Recombinant human HGF | Hepatocyte Growth Factor | 50 ng/mL | Peprotech |
| | Recombinant human Noggin | Bone Morphogenetic Protein 4 antagonist | 100 ng/mL | Peprotech, R&D Systems |
| | Recombinant human Rspondin-1 | Wnt/β-catenin signaling pathway stimulator | 500 ng/mL | Peprotech |
| *Supplements* | B27 | with or without vitamin A, Gnecco made in house without P4 | 2% | ThermoFisher |
| | EndoECM** | **0.01 mg/mL used as a soluble additive in stripped down media | 0.01 mg/mL | Contact authors |
| | Insulin-transferrin-selenium | supports metabolic pathways and mitigates ROS | 1% | ThermoFisher |
| | L-glutamine/Glutamax | Amino acid | 2 mM | ThermoFisher |
| | N2 | Gnecco made in house without P4 | 1% | ThermoFisher |
| | N-Acetyl-L-cysteine | Antioxidant and mucolytic agent | 1.25 mM | Biogems, Sigma |
| | Nicotinamide | Supplement amide derivative of vitamin B3 and a PARP inhibitor | 10 nM - 10 mM | Biogems, Sigma |
| *Inhibitors* | A83-01 | TGF-β/ALK pathway inhibitor | 500 nM - 2.3 μM | Biogems, Systems Biosciences |
| | SB202190 | p38 inhibitor | 10 μM | Sigma |
| | Y-27632*** | Rocki pathway inhibitor ***only used temporarily after passage | 10 μM | Sigma, Tocris |
| *Antibiotic/antifungal* | Penicillin/streptomycin | Anti-biotic, antifungal | 1% | ThermoFisher |
| | Primocin | Anti-biotic, antifungal | 100 μg/mL | Invivogen |
| *Hormones* | 17-β estradiol (E2) | ↑ proliferation, ESR expression | 1-10 nM | Sigma |
| | 8-Bromoadenosine 3'5'-cyclic monophosphate (cAMP) | w/ P4 accelerates ↑ glandular phenotype, ciliation | 1 μM | Sigma |
| | Human Chorionic Gonadotropin (hCG) | w/ hPL + cAMP, ↑ glandular phenotype, ↓ proliferation | 1 μg/mL | Source Bioscience |
| | Human Placental Lactogen (hPL) | w/ hCG + cAMP, ↑ glandular phenotype, ↓ proliferation | 20 ng/mL | R&D Systems |
| | Medroxyprogesterone acetate (MPA) | ↑ glandular phenotype, ↓ PGR expression, proliferation | 500 nM - 1μM | Supelco |
| | Progesterone (P4) | ↑ glandular phenotype, ↓ PGR expression, proliferation | 1 μM | Sigma |
| | Prolactin (PRL) | Secreted by decidualized stromal cells | 20 ng/mL | Peprotech |
