# Supplementary material for: WERF Endometriosis Phenome and Biobanking Harmonisation Project for Experimental Models in Endometriosis Research (EPHect-EM-Organoids): endometrial organoids as an emerging technology for endometriosis research
Source: Mol Hum Reprod. 2025 Jul 9;31(3):gaaf024. doi: 10.1093/molehr/gaaf024 (PMC12237518; doi:10.1093/molehr/gaaf024)
Supplement: gaaf024_Supplementary_Data [file gaaf024_supplementary_data.zip › MHR-24-0373-R1-SuppFileS1.pdf]

Elizabeth E. Marr, Juan S. Gnecco, Stacey A. Missmer, Shannon M. Hawkins, Kevin G. Osteen, Lone Hummelshoj, Erin Greaves, and Kaylon L. Bruner-Tran for the EPHect-Experimental Models Working Group

TABLE of CONTENTS

SOP 1: Establishment and expansions of endometrial epithelial organoids (EEOs) 2

SOP 1: Establishment and expansions of endometrial epithelial organoids (EEOs)

Purpose

To outline the establishment and propagation of primary endometrial epithelial organoid (EEO) cultures for in vitro modeling. The purpose of these EPHect SOPs is to establish a standard for practices and techniques associated with endometrial epithelial organoid culture. This SOP is designed to establish organoids from primary human tissues. Isolation and collection of endometrial epithelial glands is described in other SOPs that describe the digest, purity validation, and cryopreservation of primary epithelial glands (Boretto *et al.* 2017; Turco *et al.* 2017; Fitzgerald *et al.* 2019; Luddi *et al.* 2020).

Notes

- Proper aseptic technique prevents contamination of cultures from foreign bacteria which can spread and interfere with lab results. Safety protocols must be always maintained and proper institutional approval to work with primary human tissues must be granted prior to working with primary cells.
- Organoid are defined herein as epithelial three-dimensional (3D) gland-like structures that capture the structure and function of the endometrial epithelium and are derived from adult primary tissues cultured in a hydrogel and in a defined media composed of growth factors and inhibitors made to block specific signaling pathways such as Rho/Rock and TGFβ/ALK5.
- The experimental design and hypothesis may require modifications in hydrogels biomaterials used and/or the media formulations including additional components or different concentrations.

Supplies and equipment

- Freshly acquired human endometrial tissues, minced and in sterile PBS (See separate SOP)
    - Freshly isolated or cryopreserved endometrial epithelial gland fragments with purity of >95% from other cellular contaminants
- <u>Non-tissue</u> culture treated 24 well plates
- Pipettes tips for p10, p200, p1000 pipettes
- Cold and room temperature tips for p10, p20, p1000
- Organoid expansion medium (see below for formulation)
- Biosafety Cabinet (BSL2) for cell culture
- CO2 incubator at 37°C and 5% CO2
- Neutralizing media (DMEM/F12 + 1% penstrep, + 5% heat inactivated FBS)
- Ice bucket
- N2 supplement (Life Technologies/Fisher, Cat #: 17502048)
- B27 Life Technologies minus Vit A (Life Technologies/Fisher, Cat #: 12587010)
- recombinant Noggin 5 µg (Biogems/Peprotech, Cat #: 120-10c)
- recombinant human FGF-10 (Biogems/Peprotech, Cat #:100-26)
- A83-01 (Biogems/Peprotech, Cat #: 9094360)
- Nicotinamide (Biogems/Peprotech, Cat #: 9899208)
- N-Acetyl-L-cysteine (Sigma/Fisher, Cat #: 160280250)
- Cell Recovery Solution (Fisher Scientific, Cat #: 354253)
- Recovery Cell Freezing Medium (Life Technologies, Cat #: 12648-010)
- Dnase 1 (Sigma, Cat #: D4527-40KU)
- Dimethyl sulfoxide (DMSO) (Sigma, Cat #:D2650-100ML)
- R-Spondin. (Biogems/Peprotech, Cat #: 120-38)
- Matrigel Phenol red free (> 8.5 mg/mL conc.) (Fisher/Corning, Cat #: 356231)
- ROCK inhibitor Y-27632(Tocris, Cat #:1254)
- recombinant hEGF (Corning, Cat #: 354052)
- Insulin Transferrin Selenium (ITS-G) 100X (Life Technologies, Cat #: 41400045)

- 17-β estradiol (Sigma, Cat #: 50-28-2)
- TrypLE Express (Gibco, Cat #: 2604-021)
- Phenol red free DMEM/F12 (Gibco, Cat #: 12634-010)
- Fetal bovine serum (charcoal stripped heat inactivated) (Gibco, Cat #: 10082147)
- Penicillin/Streptomycin (Gibco, Cat #: 15140-148)
- Glutamax (Gibco, Cat #: 35050-061)

PROTOCOL

Basic principles of organoid cultures

Organoids are three-dimensional (3D) cell structures derived from stem/progenitor cell population that recapitulate the architecture and the function of their tissue of origin. These models were pioneered with the goal of developing a model to understand the stem-cell niche of organ generation and offer unprecedented capacity to give insight into epithelial biology, tissue regeneration, morphogenesis and differentiation processes. These epithelial cell cultures are genetically stable, can be expanded almost indefinitely and can be cryopreserved for long-term storage without losing great cell viability, and function.

A. Establishing cultures from cryopreserved

1. Prewarm 4 mL EEO expansion medium (Table 1).
2. Thaw cryotube of epithelial gland fragments or intact organoids in the 37°C water bath for approximately 1 min.
3. Immediately after the ice disappears, transfer the thawed cells to the prewarmed media.
4. Spin down at 300g for 5 min. Discard supernatant. Keep tube with cell pellet on ice.
5. Resuspend in Matrigel and plate 15 µL drops in center of 24-well plate well (3-4 15 µL drops per well) while keeping tips, Matrigel and tubes on ice.

   Note: *The amount of Matrigel needed for resuspension will depend on the pellet. For frame of reference an approximate number of intact organoids (20 fragments/µL) can be used for cell density.*

6. Incubate for approximately 15 mins until Matrigel solidifies and add 500 µL expansion medium with Rock Inhibitor per well.
7. Incubate the cell culture changing media every 3 days.

   Note: *Rounding of the glands should be observed after 24-48 hrs signaling the generation of organoid cultures.*

B. Passaging and dissociation of organoids to single cell

1. After the organoids have formed and have reached a diameter (>250 ums) the organoids can be collected and dissociated to single cells to expand the cultures from single cell.
2. First, remove medium from wells. Careful to not remove or aspirate the droplet.

   Note: *If the organoid/Matrigel is free floating, collection of the entire media and cells can be performed if the cells mix is spun, and the media is carefully removed and replaced with CRS. Remaining media will slow down the Matrigel removal.*

3. Add 500 µL of cold Cell Recovery Solution per well and manually detach the gel with a wide bore pipette tip making sure to fully scrape the entire well to lift off all Matrigel/organoids.
4. Use a p1000 that has been primed with Neutralizing Medium (pipetted up a few times, to avoid sticking of the organoids onto the pipette tip) and transfer the Matrigel and organoid mixture to a 15mL conical tube. Upon removal of all gel, add an additional 500 µl of Cell recovery solution to wash and collect all remaining EEOs and Matrigel fragments.

5. Place the cells and incubate 30 mins on ice to dissolve remaining Matrigel (cells should sediment to the bottom of the gel).
6. Centrifuge at 350g for 5 mins at 4°C.

   Note: *You should see a nice clean pellet, if not, then Matrigel remains and must be incubated longer and/or pipetted with p1000.*

7. Discard supernatant and add 1mL of prewarmed TypLE express with additional DNAase (10µl, 1:100) to prevent clumping. Resuspend and briskly pipette with neutralised p1000 pipette, then incubate the cells in a water bath at 37°C for 5 mins.

   Note: *Put a pipette tip on a pipettor and press against a hard surface (the sterile box) to bend the tip of the pipette. Then use the pipettor to pipette organoids. This should disrupt and break down the organoids into single cells.*

8. Pipette with a bent neutralised P1000 to break organoids into small fragments followed by a bent tip neutralized p200 (100µl).

   Note: *You might see some clumping, try to break it down by continuing to pipette. If there is a lot of clumping or a "wisping" of cells then more DNAse is needed.*

9. Repeat steps 6 and 7 by placing the tube back at 37°C at least two more times or until a single cell suspension is observed through a microscope. Continue pipetting (with neutralized bent tips) until homogenous single cell suspension – May add more TrypLE with DNase (1mL) if necessary. Neutralize the trypsin with 3 mL of neutralizing media and continue to break. Be careful of losing many cells by sticking to the pipette tips.
10. Centrifuge the cell suspension at 350g for 5 mins to pellet single cells and aspirate the neutralizing media.
11. Add up to 1 mL of full expansion medium with DNAse (1:40) and Rock inhibitor and resuspend the cell pellet. The cells should be single cells and no, or minimal clumping or wisping, should be observed.
12. Centrifuge the 15 mL tube at 350g for 5 mins to pellet single cells.
13. Collect 10 µl in a 1.5 mL centrifuge tube for cell counting.
14. Use the countess or hemocytometer to count the number of single cells to calculate the total number of cells needed for experiment or expansion of cells.
15. Cells are now ready for expansion in Matrigel.

C. Cell seeding and organoid expansion in Matrigel

1. To expand the organoid lines, single cells collected from the steps above can be re-seeded in Matrigel to expand the organoid populations.
2. Aspirate the neutralised media carefully not to disrupt or aspirate the cell pellet, leave residual media if needed, the minimal dilution of Matrigel can be around 70%). Add desired amount of ice cold Matrigel with the cold pipette tips, while keeping the cells and the Matrigel on ice. Continue to fully resuspend the cells using the COLD tips.

   Note: *Typical seeding density includes a 50-60 µl droplets with 60,000 single EEO cells. For example, if you have 700K total, then you can resuspend in 700 µl of Matrigel and seed just shy of 12 wells/droplets at* ***1K cells/µl***

3. Seed 24 well plate with 15 µL drops (3-4 per well), ensuring they stay rounded in center of wells. Incubate 15 mins at 37°C for gelation.

4. Add 500 µL pre-warmed EEO medium per well with Rock inhibitor if not already added.
5. Feed subsequently every 3 days and passage as necessary.
6. Check the plates frequently, the EEOs should be beginning to get round after second day. Clumps of cells will tend to grow faster and better than single cells.

   Note: *In EEOs, ROCK inhibitor (ROCKi) is temporarily added to media during expansion and passaging, but then removed for long term maintenance. This inhibitor is crucial in the first 3-4 days of culture to increase epithelial cell viability but can be removed after cells have begun to divide.*

D. Freezing

1. Remove medium from wells.
2. Perform EEO expansion protocol (above) to get a pellet of intact organoids.

   Note: *Freezing as intact organoids, like fragments, increases the viability and recovery upon thawing.*

3. Resuspend to a concentration of 2000 EEOs per ml (by cell counting) or 1 mill cells/mL in Cell Recovery Freezing Medium.
4. Transfer 1 mL of cell concentrations into liquid nitrogen cryogenic tubes.
5. Place tubes in slow freeze cooler overnight at -80C.
6. Transfer tubes to liquid nitrogen storage the following day.

Figure 1: Workflow for the establishment and expansion of epithelial organoid lines

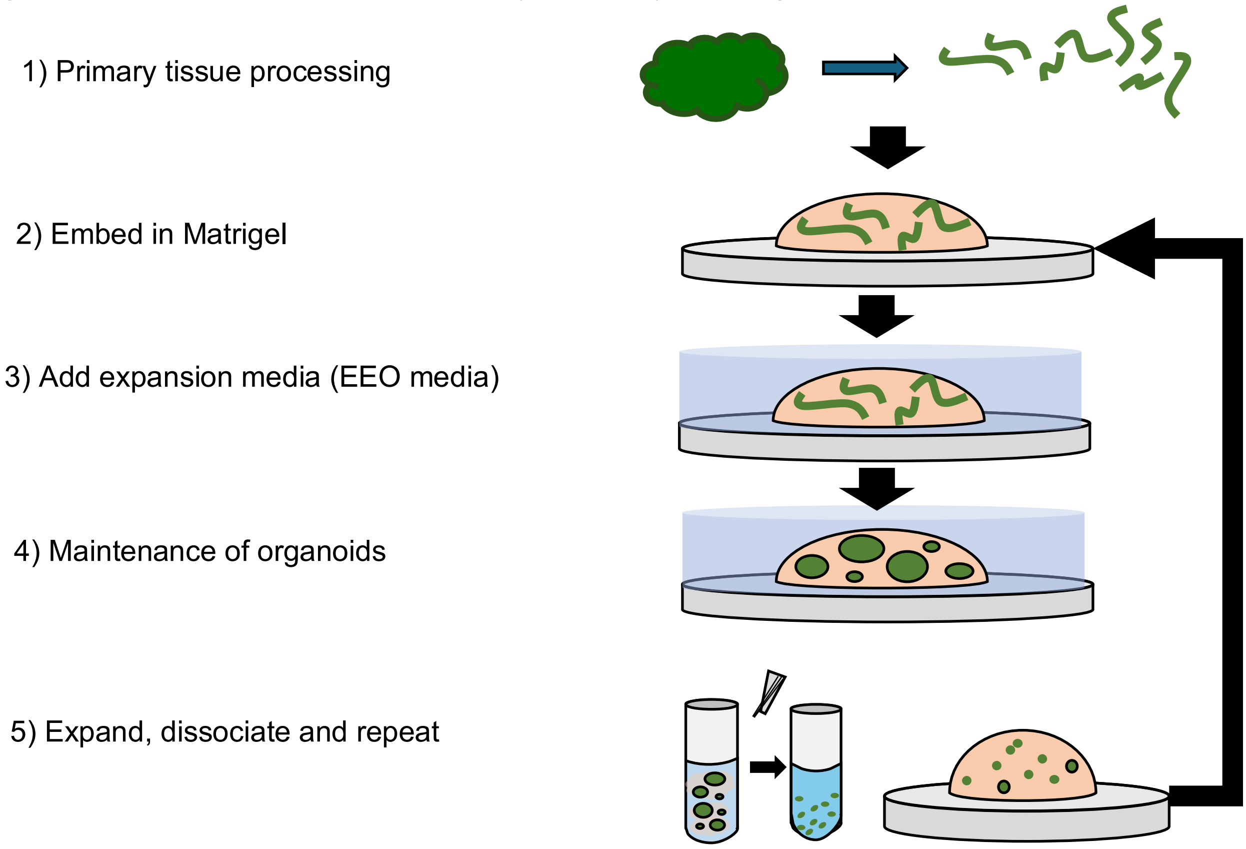

Table 1.1 EEO expansion media

COMPONENT	VOLUME IN 50 ML	STORAGE	STOCK CONCENTRATION	FINAL CONCENTRATION
DMEM/F12	29 mL	4°C	N/A	N/A
Advanced Basal Media (ABM)*	20 mL	4°C	2.5x	1x
ITS	500 µL	4°C	100x	1%
rhEGF	50 µL	-20°C	50 µg/mL	50 ng/mL
rhNoggin	50 µL	-20°C	100 µg/mL	100 ng/mL
A8301	50 µL	-20°C	500 µM	500 nM
Nicotinamide	50 µL	-20°C	1 M	1 mM
N-Acetyl-L-Cysteine	50 µL	-20°C	1.25 M	1.25 mM
Estradiol	50 µL	4°C	1 µM	1 nM
rhFGF-10	25 µL	-20°C	100 µg/mL	50 ng/mL
rhRspondin-1	10 µL	-20°C	1 mg/mL	200 ng/mL
**OPTIONAL**				
Rock Inhibitor	50 µL	-20°C	10 mM	10 µM
Progestin (MPA)	50 µL	-80°C	500 µM	500 nM
PROGESTERONE (P4)	10 µL	4°C	0.5 mM	100 nM

Table 1.2 Advanced Basal Media (2.5x Concentrated)

COMPONENT	VOLUME IN 40 ML	STORAGE	STOCK CONCENTRATION	FINAL CONCENTRATION
DMEM/F12 (phenol-free)	35 mL	4°C	N/A	N/A
B27 Supplement – Vit A	2 mL	-20°C	50x	2.5x
N2 Supplement	1 mL	-20°C	100x	2.5x
Pen Strep	1 mL	4°C	100x	2.5x
Glutamax	1 mL	4°C	100x	2.5x

Note: *The described media supplements N2 and B27 are known to contain progesterone as a component of the original formulation (Brewer et al., 1993; Brewer, 1995).*

DEFINITIONS

PBS -/-: PBS without calcium and magnesium
BSC: Biosafety cabinet
EEO: Endometrial Epithelial Organoids
EEO expansion medium: See media

REFERENCES

Boretto M, Cox B, Noben M *et al.* Development of organoids from mouse and human endometrium showing endometrial epithelium physiology and long-term expandability. *Development* 2017;144:1775–86.

Brewer GJ. Serum-free B27/neurobasal medium supports differentiated growth of neurons from the striatum, substantia nigra, septum, cerebral cortex, cerebellum, and dentate gyrus. *J Neurosci Res* 1995;42:674–83.

Brewer GJ, Torricelli JR, Evege EK *et al.* Optimized survival of hippocampal neurons in B27-supplemented Neurobasal, a new serum-free medium combination. *J Neurosci Res* 1993;35:567–76.

Fitzgerald HC, Dhakal P, Behura SK *et al.* Self-renewing endometrial epithelial organoids of the human uterus. *Proc Natl Acad Sci* 2019;116:23132–42.

Luddi A, Pavone V, Semplici B *et al.* Organoids of Human Endometrium: A Powerful In Vitro Model for the Endometrium-Embryo Cross-Talk at the Implantation Site. *Cells* 2020;9:1121.

Turco MY, Gardner L, Hughes J *et al.* Long-term, hormone-responsive organoid cultures of human endometrium in a chemically defined medium. *Nat Cell Biol* 2017;19:568–77.
